# Multiple Advantageous Amino Acid Variants in the *NAT2* Gene in Human Populations

**DOI:** 10.1371/journal.pone.0003136

**Published:** 2008-09-05

**Authors:** Francesca Luca, Giuseppina Bubba, Massimo Basile, Radim Brdicka, Emmanuel Michalodimitrakis, Olga Rickards, Galina Vershubsky, Lluis Quintana-Murci, Andrey I. Kozlov, Andrea Novelletto

**Affiliations:** 1 Department of Cell Biology, University of Calabria, Rende, Italy; 2 Institute for Haematology and Blood Transfusion, Prague, Czech Republic; 3 Institute of Molecular Biology and Biotechnology, Heraklion, Greece; 4 Department of Biology, University “Tor Vergata”, Rome, Italy; 5 Arct. An. C Innovative Laboratory, Moscow, Russia; 6 Institute of Developmental Physiology, Russian Academy of Education, Moscow, Russia; 7 UP Human Evolutionary Genetics, CNRS-URA3012, Institut Pasteur, Paris, France; University of Glasgow, United Kingdom

## Abstract

**Background:**

Genetic variation at *NAT2* has been long recognized as the cause of differential ability to metabolize a wide variety of drugs of therapeutic use. Here, we explore the pattern of genetic variation in 12 human populations that significantly extend the geographic range and resolution of previous surveys, to test the hypothesis that different dietary regimens and lifestyles may explain inter-population differences in *NAT2* variation.

**Methodology/Principal Findings:**

The entire coding region was resequenced in 98 subjects and six polymorphic positions were genotyped in 150 additional subjects. A single previously undescribed variant was found (34T>C; 12Y>H). Several aspects of the data do not fit the expectations of a neutral model, as assessed by coalescent simulations. Tajima's D is positive in all populations, indicating an excess of intermediate alleles. The level of between-population differentiation is low, and is mainly accounted for by the proportion of fast vs. slow acetylators. However, haplotype frequencies significantly differ across groups of populations with different subsistence.

**Conclusions/Significance:**

Data on the structure of haplotypes and their frequencies are compatible with a model in which slow-causing variants were present in widely dispersed populations before major shifts to pastoralism and/or agriculture. In this model, slow-causing mutations gained a selective advantage in populations shifting from hunting-gathering to pastoralism/agriculture. We suggest the diminished dietary availability of folates resulting from the nutritional shift, as the possible cause of the fitness increase associated to haplotypes carrying mutations that reduce enzymatic activity.

## Introduction

Arylamine N-acetyltransferases (NATs) are drug-metabolizing enzymes that catalyze the conjugation of an acetyl group from acetyl CoA onto an amine, hydrazine or hydroxylamine moiety of an aromatic compound [1]. In humans, duplicated genes encode for two enzymes (NAT1 and NAT2) with distinct, but partially overlapping substrate specificities and with different tissue distribution: NAT1 is virtually ubiquitous, whereas NAT2 is expressed at high levels only in the intestine and liver [2]. NAT1 specific substrates include *para*-aminobenzoate (PABA), 4-aminosalicylic acid and *para*-aminobenzoylglutamate (pABGlu, a folate catabolite) [3]–[5]. NAT2 is active on a wide range of substrates, but particularly arylhydrazine compounds, thus providing a major route to detoxification.

Genetic variation at *NAT2* has been long recognized as the cause of differential ability to metabolize a wide variety of drugs of therapeutic use in diseases with high prevalence such as tuberculosis, arrhythmia and hypertension [6]. In fact, three major phenotypes are observed, i.e. the fast, intermediate and slow acetylators, which are inherited as a codominant trait. It is also recognized that the different activity of the allelic isoenzymes results in altered activation/deactivation of many compounds that are commonly found in native food or are generated by cooking or other treatments. Additionally, depending on the metabolites that are generated and on the body compartments where they are dispatched, the products of NAT2 activity can exert carcinogenic or toxic effects [7].

**Figure 1:**
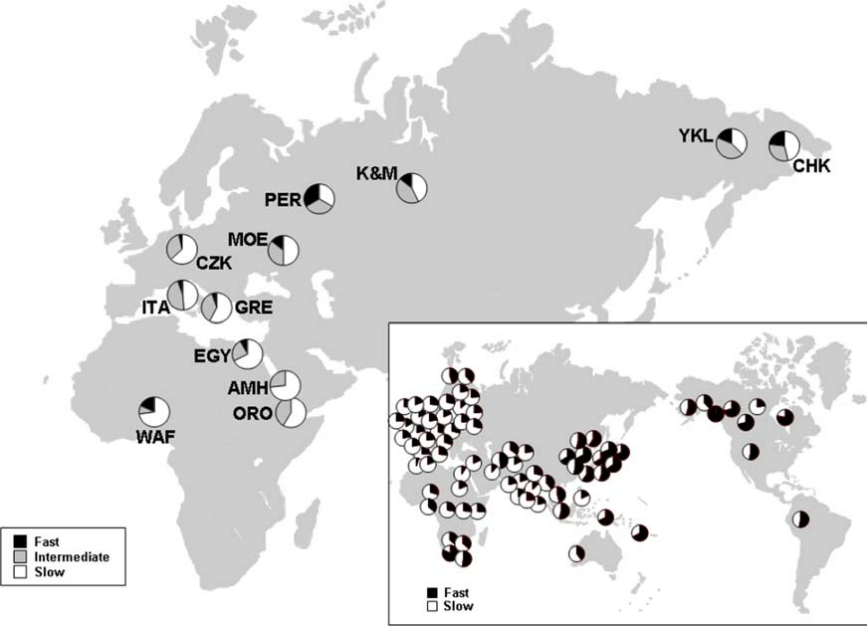
Map of the old world showing the sampling locations for the populations here examined. Each pie represents the percentage of the slow (white), intermediate (grey), and fast (black) acetylators in the extended panel, inferred from genotype data as described in Materials and Methods. Inset: map showing the frequency of slow (white) and fast (black) haplotypes as inferred based on biochemical data [6].

Thanks to the well-established genotype-phenotype correlation, the overall population prevalence of the acetylator phenotype can be inferred from the population frequency of variants known to affect NAT2 function. Because of its medical interest, the geographic distribution of *NAT2* variants has been well characterized. This distribution shows a belt of populations with high frequencies of the slow-acetylator allele(s) stretching from Europe to central and northern Africa to southern Asia; in constrast, populations from southern Africa, eastern Asia and the Americas are characterized by higher frequencies of the fast acetylator allele(s) (Fig. 1, data from [6], [8] and references therein). Wilson et al. [9] analysed *NAT2* and other genes coding for drug metabolizing enzymes and found greater heterogeneity among genetically-defined than ethnically-defined populations. This finding suggests that a population's drug-metabolizing repertoire is likely to vary across local populations within broadly defined human groups. These findings raise the question of whether the current geographic distribution of *NAT2* variants is the result of human adaptations to different and changing environments. It is implicit that such adaptations were induced by long-term exposure to environmental chemicals and dietary components rather than medical drugs, which were introduced too recently to result in any considerable selective effect.

Here, we report on the pattern of genetic variation at *NAT2* coding region in 12 human populations that significantly extend the geographic range (Fig. 1) and resolution of previous *NAT2* surveys [8], [10]–[12]. In this study, populations were selected according to two criteria: i) they represent a wide spectrum of environmental and dietary regimens that may have shaped *NAT2* variation and ii) they represent test samples of large national communities that may become exposed to drugs metabolized by NAT2 isoenzymes for the treatment of a number of diseases of major Public Health importance (e.g. tuberculosis). Moreover, the ongoing westernization of the life style and diet of these same communities e.g. [13] is likely to modify substantially their cancer risk, which is also a function of their *NAT2* genotypic pool. Our results show that spatially and temporally varying selection reflecting different dietary regimens and lifestyles may explain inter-population differences in *NAT2* variation.

## Results

Twelve population samples were included in our study of *NAT2* variation; these populations cover a geographic area extending from Africa North of the equator to South and East Europe and North East Asia to the Beringian coast. The extremes of the geographic range include three African populations (Dendi from Nigeria and the Amhara and Oromo from Ethiopia) and a Siberian population from the Chukotka peninsula. We selected small sub-samples (6–14 individuals) from 9 of the 12 above sampling locations for a full re-sequencing survey (referred to as “re-sequencing panel”, see Supplementary Table S1). Also, seven known polymorphisms were genotyped in additional 150 individuals from the same 12 populations, bringing the overall number of genotyped subjects to 248 (referred to as “extended panel”, see Supplementary Tables S1 and S2).

### Re-sequencing survey

The *NAT2* gene spans 9.9 kb (Chr 8 positions 18,293,034–18,302,961 in NCBI B35 assembly) and consists in a non-coding exon at the 5′ end, separated by a 9 kb intron from a uninterrupted coding region of 873 bp (including the stop codon). Because a previous study had reported a putative signal of non-neutral evolution for the *NAT2* coding region only [8], we re-sequenced this region in a total of 98 subjects from nine populations (listed in Supplementary Table S2), thus significantly extending the geographic range and resolution of previous surveys. This allowed the search for possible, yet undetected, variants. A single new variant was found (34T>C); it results in a non-synonymous change (12Y>H) whose effects on NAT2 function are unknown. This variant was found in a Chukchee individual homozygous for two haplotypes otherwise identified as *5B. This polymorphism was only considered in the neutrality tests and when calculating Extended Haplotype Homozygosity (EHH).

The remaining 6 polymorphic sites are the ones most commonly reported in surveys of *NAT2* variation in human populations (http://www.louisville.edu/medschool/pharmacology/NAT.html). Two are synonymous changes (C282T and C481T), whereas the remaining four variants are non-synonymous (T341C, G590A, A803G and G857A). Although G191A is described to be relatively common in Western Africa (up to 15%) [8], [12], [14], none of the subjects in our sample carried this variant.

The ancestral state at each variable position was inferred by comparison with the chimpanzee and macaque sequences; both outgroup sequences showed the same nucleotide at each polymorphic position in humans. We find that haplotype *4 carries the ancestral allele at all variable sites detected in our survey as well as at all the polymorphisms reported in the *NAT2* literature, thus identifying this fast haplotype as the ancestral arrangement of human polymorphic alleles.

Extensive theoretical work [15]–[20] has shown that surveys of ascertained SNPs may introduce a bias in the distribution of allele frequencies, linkage disequilibrium and polymorphism levels and, therefore, affect population genetics inferences. In contrast, full re-sequencing surveys, in which the sequence of a genomic segment is determined in each individual in the sample, allow an unbiased characterization of multiple aspects of genetic variation and are suitable for standard methods of population genetics analyses. In this sense, the set of 196 (98×2) gene copies entirely sequenced for the *NAT2* coding region provides an appropriate data set for performing statistical tests based on a variety of summaries of genetic variation data. For this survey, we focused on the portion of the gene for which a precise genotype/phenotype correlation has been established [7]; however, we cannot rule out that non-coding variants exist, which influence *NAT2* gene expression and function. Polymorphism levels were summarized by the estimators Theta(pi) and Theta(s), which are based on average number of pairwise sequence differences and on the number of segregating sites, respectively. In the three continental pools, the estimator Theta(pi) exceeded the estimator Theta(s) (Table 1). Under the standard neutral model, the distributions of the two parameters have the same mean. While chance variation predicts that, in any given sample, one of the parameters may exceed the other, Theta(pi) also exceeded Theta(s) in all individual populations samples (Supplementary Table S3). Consequently, Tajima's D [21], a summary statistic of the frequency spectrum based on the difference between the Theta estimators, was invariably positive in the continental and in the individual population samples, indicating the excess of intermediate frequency alleles as a feature of *NAT2* variation.

**Table 1:** Summary statistics of *NAT2* diversity in the continental samples of the resequencing panel.

Population	N^1^	Hd^2^	Theta(pi)%	Theta(pi)% (Syn)	Theta(pi)% (NSyn)	Theta(s)%	Theta(s)% (Syn)	Theta(s)% (NSyn)	TD^3^	Fay&Wu H
Africa	32	0.562	0.251	0.439	0.197	0.142	0.250	0.111	2.050** ^̂^##^	−0.456
Europe	88	0.768* ^̂^##^	0.267	0.465	0.210	0.136	0.200	0.118	2.213** ^̂^##^	+0.899*^##^
Asia	76	0.829*** ^̂̂^###^	0.271	0.455	0.218	0.164	0.206	0.152	1.607* ^	+1.396* ^̂̂^###^
All	196	0.775	0.273	0.465	0.217	0.137	0.172	0.127	2.110	+1.027

1. Number of gene copies; 2.Haplotype diversity; 3. Tajima's D.

Neutrality test significance under demographic model (see Materials and Methods):

Exponential growth, ^*^0.1<P<0.05, ^**^P<0.05, ^***^P<0.01.

Bottleneck A, ^0.1<P<0.05, ^̂P<0.05, ^̂̂P<0.01.

Bottleneck B, ^#^0.1<P<0.05, ^##^P<0.05, ^###^P<0.01.

Table 1 also reports the statistical significance of the departure of summary statistics from null distributions obtained under three commonly considered demographic models (see Materials and Methods). When compared to the results of neutral simulations under the exponential growth model: Tajima's D values were significantly positive for the African and European samples (p<0.05) and reached borderline significance in the Asian sample; Fay and Wu's H reached borderline significance in the European and Asian samples. When compared to the results of neutral simulations under the Bottleneck model A: Tajima's D values were significantly positive for the African and European samples (p<0.05) and reached borderline significance in the Asian sample; Fay and Wu's H was significant in the Asian sample (p<0.01). When compared to the results of neutral simulations under the Bottleneck model B: Tajima's D values were significantly positive for the African and European samples (p<0.05); Fay and Wu's H was significant in the European (p<0.05) and Asian (p<0.01) samples.

The non-synonymous variants (T341C, G590A, A803G and G857A) make a substantial contribution to the overall diversity, as their Theta(pi) is also always greater than Theta(s) in all Continents (Table 1, cols. 6 vs 9). These results did not change when samples were considered analytically, indicating that the non-synonymous variable sites reaching intermediate frequencies are in excess, reproducibly across populations (Supplementary Table S3).

Additionally, both the haplotype count (not shown) and haplotype diversity (Table 1) in Asian and European populations significantly exceeded those obtained from the coalescent simulations under neutral scenarios, assuming the rho value estimated from the HapMap data for the *NAT2* genic region (0.24).

The partitioning of sequence diversity according to lineages marked by slow-causing mutations was very informative. The average number of nucleotide differences between pairs of sequences (formally identical to Theta(pi) per gene) in the pool of fast haplotypes was by far the highest (0.75). Conversely, it turned out to be much lower for the *5, *6 and *7 series (0.13, 0.06 and 0.24, respectively).

We also investigated the haplotype structure in the *NAT2* region by calculating the EHH [22] on both sides of each slow-causing mutation (Supplementary Fig. S2a, top line). In all cases, the derived allele at positions 341, 590 and 857 showed higher EHH than the ancestral allele. This difference turned out not to be significant when tested against simulations under a standard neutral model.

In order to verify this finding using data from a larger genomic region, we repeated the same analysis using the HapMap Phase II data for more than 200 SNPs spanning approximately 100 kb centered on the *NAT2* coding region [23]. The results showed higher EHH for haplotypes harbouring the derived (slow-causing) allele at positions 341, 590 and 857 in all HapMap populations on both sides of these positions (Supplementary Fig. S2b–d, top lines). Similar results were obtained also for the G191A in Yorubans (Supplementary Fig. S2b). Qualitatively, EHH profiles were remarkably similar between Yoruba and the other populations, more so for fast haplotypes. Taken at face value, this indicates that non-African populations did not suffer any major bottleneck.

The majority of the common variants are non-synonymous (5 vs. 2 synonymous) and are known to reduce NAT2 activity to a similar extent. Though this proportion is not statistically significant in the McDonald-Kreitman test (9 vs. 3 fixed differences between human and chimpanzee), we notice that this test does not take into account the frequency of each variant. Instead, our data indicate that considering allele and haplotype frequencies is crucially important.

Thus, we provisionally conclude that multiple slow acetylator mutations contribute an excess polymorphism to the *NAT2* coding sequence. Even though neutral evolution cannot be unequivocally excluded, the departure of the observed frequency spectrum from the simulated neutral scenarios, coupled with the presence of multiple non-synonymous variants with well documented phenotypic effects, suggests that positive natural selection drove these variants to intermediate frequencies at some point during their evolutionary history. Our resequencing data do not allow us to distinguish between different selective scenarios (e.g. directional selection on multiple standing variants vs. balancing selection).

### Genotyping data

The 6 common polymorphic variants identified in the resequencing panel were also genotyped in an additional set of 150 individuals (for a total of 248 individuals) (Supplementary Tables S1 and S2). Table 2 displays the structure of haplotypes as well as their population frequencies.

**Table 2:** Relative haplotype frequencies in 12 population samples.

	282	341	481	590	803	857	Activity	Dendi	Amhara	Oromo	Egyptians	Italians	Greeks	Czechs	Mordvin	Russian	Khanty Mansi	Yakuts	Chukchee
	Syn	N-s	Syn	N-s	N-s	N-s													
N (subjects)								11	15	12	37	37	40	27	14	12	14	16	13
Macaque	C	T	C	G	A	G													
Chimpanzee	C	T	C	G	A	G													
Haplotype																			
NAT2*4	.	.	.	.	.	.	F	0	0.06	0.08	0.12	0.24	0.21	0.2	0.21	0.33	0.14	0.38	0.35
NAT2*5°	.	**C**	T	.	.	.	S	0	0	0.04	0.01	0	0.01	0	0	0.04	0	0.03	0
NAT2*5B	.	**C**	T	.	G	.	S	0.23	0.53	0.33	0.49	0.32	0.38	0.39	0.50	0.33	0.29	0.19	0.19
NAT2*5C	.	**C**	.	.	G	.	S	0.05	0	0.04	0.01	0.03	0.04	0.06	0	0	0.07	0	0
NAT2*5D	.	**C**	.	.	.	.	S	0.05	0	0	0	0	0	0.06	0	0	0	0	0
NAT2*5G	T	**C**	T	.	G	.	S	0.05	0.03	0	0	0	0	0	0	0	0	0	0
NAT2*5J	T	**C**	.	**A**	.	.	S	0	0	0	0	0	0	0.04	0	0	0	0	0
NAT2*6°	T	.	.	**A**	.	.	S	0.27	0.27	0.21	0.27	0.34	0.29	0.26	0.11	0.08	0.11	0.25	0.27
NAT2*6B	.	.	.	**A**	.	.	S	0.09	0	0	0	0	0.01	0	0	0	0	0	0
NAT2*6C	T	.	.	**A**	G	.	S	0.05	0	0	0	0	0	0	0	0	0	0	0.04
NAT2*7°	.	.	.	.	.	**A**	S	0	0	0	0	0	0.01	0	0	0	0	0	0.04
NAT2*7B	T	.	.	.	.	**A**	S	0	0.03	0.17	0.01	0.03	0.03	0	0.07	0.04	0.18	0.13	0.08
NAT2*11°	.	.	T	.	.	.	F	0	0	0	0	0.01	0	0	0	0	0.11	0	0
NAT2*12°	.	.	.	.	G	.	F	0.05	0.03	0.08	0.01	0	0	0	0	0.08	0.04	0	0.04
NAT2*12B	T	.	.	.	G	.	F	0.05	0	0	0	0	0	0	0	0.04	0	0	0
NAT2*12C	.	.	T	.	G	.	F	0	0	0	0	0.01	0.01	0	0.07	0	0.04	0.03	0
NAT2*13	T	.	.	.	.	.	F	0.14	0.03	0.04	0.07	0.01	0.01	0	0.04	0.04	0.04	0	0

Allele states at 6 variable nucleotide positions are reported in cols. 2–7. A dot indicates identity with the ancestral state. Alleles associated with reduced activity are reported in boldface. Haplotype classification (Fast/Slow) is reported in col. 8.

Five of the 6 genotyped SNPs are polymorphic in all samples, whereas position 857 was polymorphic in all samples but the Dendi and the Czech.

Only two haplotypes are present in all population samples and both are derived, i.e. *5B and *6A. The first one exhibits frequencies above 18% in all the examined populations. The ancestral haplotype *4 is the third most common, with frequencies ranging between 0% and 38%.

Figure 1 and the Supplementary Table S1 list the phenotypes in each population sample divided into fast, intermediate and slow acetylators, inferred as described above. The results are in agreement with previous observations, by showing a belt of populations characterized by the high prevalence of slow and intermediate acetylators stretching from Eastern Africa to North-Western Europe. In contrast, a higher prevalence of fast acetylators is observed in the single Western African sample examined here and in the Northern Eurasian samples.

**Figure 2:**
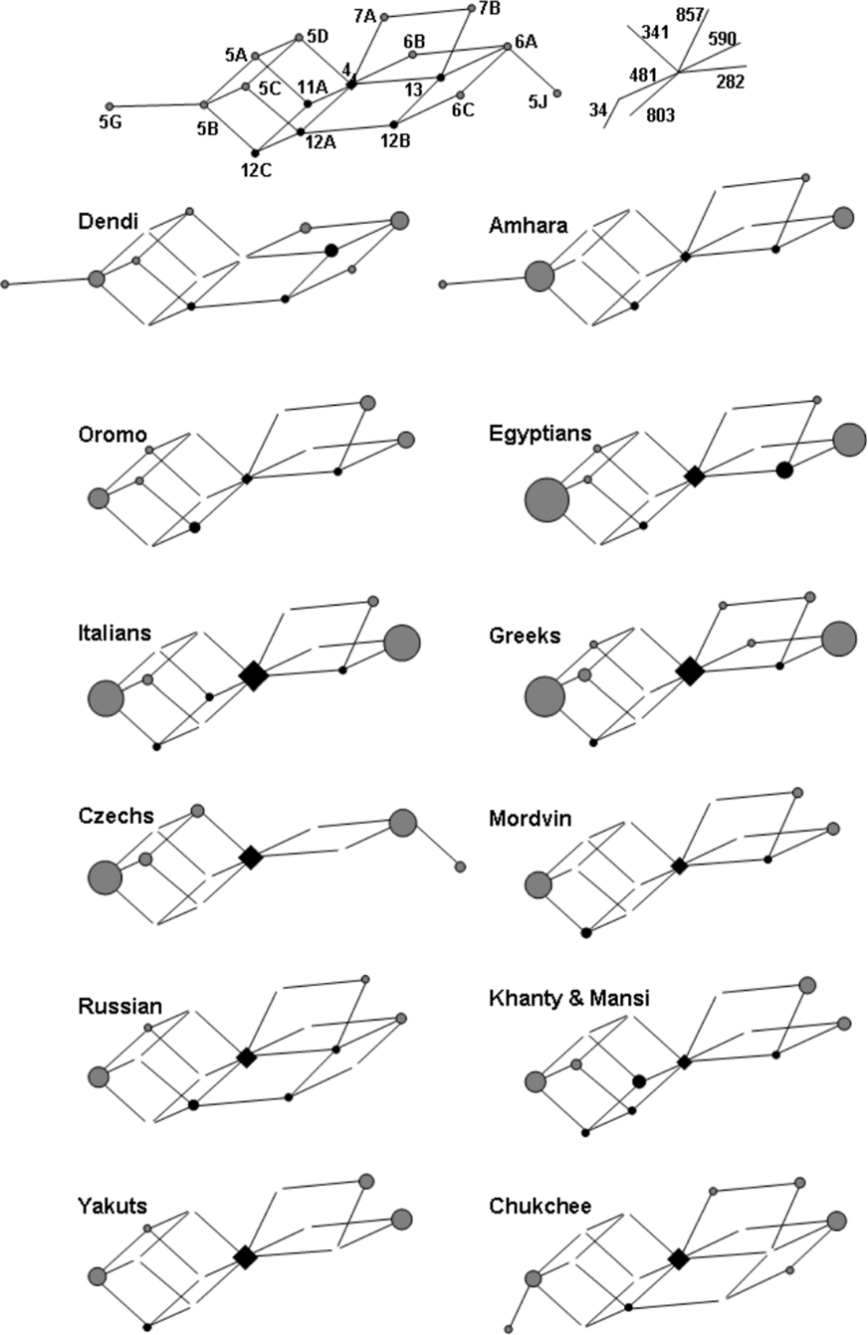
Median-joining networks of *NAT2* haplotypes in the 12 populations of the extended panel. The relationships between the 17 haplotypes found in the pool of the 12 populations are displayed by the network on top, with nodes of fixed size. Vectors representing each mutation are oriented as shown on top right (e.g. the vector 341 connects haplotypes which differ only at the position 341). Nodes representing fast and slow haplotypes are color-coded in black and grey, respectively. Networks specific for each population are displayed using the same skeleton and omitting only unnecessary links. Node size is proportional to the number of observations. Note that three main features of the overall network are necessary to explain the haplotypic composition of all but two populations (the left-hand “cube” leading to *5B and the right-hand “planes” leading to *7B and *6A), and that in most cases the median vectors of these structures are actually found. In all graphs the ancestral haplotype *4 is identified by a lozenge.

The genealogical relationships among haplotypes inferred by network analysis (Fig. 2), reveal three main features. First, the haplotypes are connected in a very condensed and highly reticulated network (Fig. 2, top). It is worth noting that the two most divergent haplotypes (*5B with 34T>C vs. *6A) differ by 6 substitutions, i.e. only 2.5 fold the average pairwise distance (2.4). When large, this measure is taken as an indicator of deep-rooted gene genealogies, possibly maintained by long-term balancing selection [24]. Second, the networks obtained for each separate population are very similar. Third, all networks show relatively common haplotypes at nodes that are two (for *6A) and three (for *5B) steps apart from the ancestral *4 haplotype, while haplotypes at intermediate nodes are present in most populations, but occur at lower frequencies. These results show that, even though we surveyed geographically diverse populations, we did not find populations harbouring common haplotypes that are ancestral to those most represented in contemporary populations, and argue for a recent increase in frequency of the terminal haplotypes. It is worth noting that, based on the network reconstruction, the three key mutations determining the slow acetylator haplotypes (T341C, G590A and G857A) tend to be more recent than mutations that do not modify the acetylator status.

Pairwise LD (Supplementary Table S4) diplays strong D' but quite low r^2^ values. In fact, no two variants are uniquely associated in a single haplotype (condition to have r^2^ = 1) and several combinations show the presence of all 4 haploid arrangements in 2×2 comparisons (e.g. 590G>A vs. 803A>G). This data are consistent with a relevant role of recombination (or other inter-chromosomal mechanisms) in reshuffling alleles at different but very closely spaced positions.

We then sought to further test the consistency of our data with a scenario of a small population exiting and expanding out of Africa, the emergence of new variants during this process and the increase of their frequencies up to intermediate values by neutral processes. We reasoned that, under this hypothesis, increasing levels of resolution (i.e. when considering phenotypes, haplotypes and haplotypes with their molecular distances) should lead to increasing values of the corresponding fixation index, due to the ongoing molecular radiation paralleling populations splits and dispersals. We then measured the intra- and inter-population quotas of genetic diversity by considering different properties of *NAT2* haplotypes. When analysed individually, all of the 6 variable positions revealed low *F*_ST_ values, with only A857G reaching 0.04 (Supplementary Table S5). At the haplotypic level, when only two groups were considered, i.e. fast and slow haplotypes (Table 3, col. 3), we obtained the lowest values for both intra- and inter-population variances. The corresponding fixation index, *F*_ST_, was 1.96%, indicating a fairly modest differentiation among the set of widely dispersed populations. When all 17 haplotypes were considered, without taking into account their actual molecular diversity (number of mutations differentiating them), larger variance components were obtained, as expected. Nevertheless the *F*_ST_ index was only slightly increased (Table 3, col. 4). Finally, when molecular distances were considered in the calculation (i.e. using Φ_ST_ in the AMOVA framework [25]), variance components reached the highest values, but the fixation index was again not increased (Table 3, col. 5). In the three conditions, the comparison between African and Eurasian populations contributed 2/3 to 1/3 of the overall inter-population diversity (not shown).

**Table 3:** Measures of intra- and inter-population diversity by using different inter-haplotypic distances.

Source of variation	d.f.	Fast vs. Slow haplotypes	17 haplotypes	17 haplotypes and molecular distance
Among populations	11	0.0039	0.0085	0.0196
Within populations	484	0.1941	0.3734	1.1834
Fixation index		Fst = 0.0196	Fst = 0.0223	Φst = 0.0163
Significance		P = 0.049	P = 0.001	P = 0.029

Variance components are reported in the body of table.

### Nutritional data

Previous studies [8] proposed a selection in favour of 341(T), which leads to a slow acetylator phenotype, as a consequence of the shift in modes of subsistence and lifestyle in human populations. Based on the results presented here and the similarity of the phenotype produced by slow-causing haplotypes, this hypothesis can be extended to all of the three slow variants. To test this hypothesis more directly, we assigned 47 populations to one of the major subsistence modes, and performed tests for the equality of haplotype frequencies across subsistence modes (Table 4).

**Table 4:** Test of equality of frequency of 5 *NAT2* haplotype series among three major modes of subsistence.

Series	Subsistence	N	Avg. Freq. (%)	p(Kruskal Wallis test)
All Fast	Hunter-gath.	12	52.1	<.001
	Pastoralist	13	35.6	
	Agriculturalist	22	27.3	
*14	Hunter-gath.	12	4.2	.006
	Pastoralist	13	0.0	
	Agriculturalist	22	1.4	
*5	Hunter-gath.	12	23.0	.017
	Pastoralist	13	27.3	
	Agriculturalist	22	37.2	
*6	Hunter-gath.	12	10.6	<.001
	Pastoralist	13	23.3	
	Agriculturalist	22	30.0	
*7	Hunter-gath.	12	10.2	.002
	Pastoralist	13	13.7	
	Agriculturalist	22	4.0	

The pool of fast haplotypes showed a strong decreasing trend in the order hunter-gatherers/pastoralists/agriculturalist. Frequency heterogeneity among the categories was significant by the non-parametric Kruskal Wallis test. Though haplotype frequencies violate the assumption of independence (due to their summing to 1), it is important to note that both *5 and *6 show a definite increase among agriculturalists, with a trend significantly departing from equality.

The vast geographic distance between populations with the same subsistence modes (especially H-G, Supplementary Figure S3) makes it unlikely that these results are due to spatial clustering and preferential gene flow. The evidence regarding the *7 haplotype, which shows the highest frequency among pastoralists, must be considered cautionarily, due to the overrepresentation of the closely spaced Central Asians.

## Discussion

### Unusual features of *NAT2* polymorphism

Our resequencing survey of *NAT2* variation in geographically dispersed human populations showed that several aspects of the data do not fit the expectations of a neutral model. This finding, coupled with the presence of multiple amino acid polymorphisms that are widely distributed and that are known to have similar phenotypic effects, suggests that these variants were advantageous and that they increased in frequency in parallel in different human populations.

Several aspects of sequence variation data at the *NAT2* gene are unusual when compared to neutral expectations for demographic models that were previously shown to fit genome-wide patterns of variation. For example, while Sub-Saharan African populations generally tend to have a genome-wide excess of rare variants which probably reflects a history of recent growth, the frequency spectrum at *NAT2* shows a marked excess of intermediate frequency variants (positive Tajima's D), which departed significantly from a neutral model of a population at equilibrium followed by rapid exponential growth. A similar excess of intermediate frequency variants was observed in non-African populations, which tend to have positive Tajima's D values at the genome-wide level; yet, the Tajima's D value at *NAT2* is unusual in the European and Asian samples (though only marginally significant in the latter) when compared to neutral expectations for two different bottleneck models. Significantly positive figures of Tajima's D underline the excess of high frequency polymorphisms and the paucity of singletons. As it is not computationally feasible to test all the space of possible demographic scenarios, this result may still be compatible with the hypothesis of a purely demographic affect, i.e. a bottleneck experienced by all of the populations here scored.

Moreover, our results depict a pattern of variation at *NAT2* very discrepant from the average of 313 and 132 genes scored by Stephens et al. [26] and Akey et al. [27], respectively. The density of SNPs/kb is large (8.03 vs. 3.4), with a Theta(s) value of the same magnitude but a very different Theta(pi) (0.273% vs. 0.034%). Based on Theta(pi), *NAT2* ranks among the most polymorphic genes as compared to other drug metabolizing genes [28]. Generalized and strongly enhanced mutation rates at *NAT2* can be excluded as Theta(s), which is an estimator of 4N_e_μ (where N_e_ is the population effective size and μ the mutation rate), falls into the range reported by Stephens et al. [26] for all genic regions. However, our results cannot rule out site-specific mutation proneness.

Also, there is a preferential polymorphism at non-synonymous sites. Six variants have intermediate frequencies above 5% worldwide, four of which are non-synonymous (non-synonymous/synonymous ratio = 2), contrasting with the findings by Wong et al. [29] for SNPs with Minor Allele Frequency (MAF) above 6.1% (non-synonymous/synonymous ratio ∼0.5). The percentage of synonymous substitutions is only two thirds of that reported by Stephens et al. [26](2/7 not conditioned on frequency vs. 459/1033). We indeed measured the dN/dS ratio in a tripartite tree including human, chimp and macaque sequences (rodents as outgroup) and found this ratio largely increased in *NAT2* (0.71) as compared to *NAT1* (0.28). As a minimal interpretation, this is in agreement with a long-lasting relaxation of selection on *NAT2*. However, the observation that 3 out of the 4 human polymorphic non-synonymous substitutions are also responsible for NAT2 slow acetylation activity (slow-causing variants) support the hypothesis that this could be an adaptive trait subjected to some form of selection in the recent past or even today. Network analysis turned out to be a powerful tool to indicate that haplotypes *5B, *6A and *7B stem out from low-frequency haplotypes radiating from the ancestral *4, which is still today an intermediate frequency haplotype. This is a pattern representing an obvious departure from the shape expected under neutrality and is responsible for the excess of positive Tajima's D values and the maintenance of longer EHH on slow haplotypes. These features can be hardly perceived if examining only numerical data such as linkage disequilibrium (LD) estimators (Supplementary Table S4).

Finally, low fixation indexes over a transept crossing the Old World, place *NAT2* at one edge of the genome-wide distribution [30]. Our results can be summarized and interpreted as follows: 1) the *NAT2* coding region is poorly differentiated in the population samples examined (absolute values of the fixation indexes); 2) the major determinant of inter-population diversity are the phenotypic proportions (the first fixation index value in Table 3 is not lower than the others); 3) population dispersals were not accompanied by a concomitant accumulation of molecular diversity (similarity between the 2^nd^ and 3^rd^ fixation index); 4) the data fit the distribution obtained by Currat et al. [31] for neutral alleles already attaining polymorphic frequencies at the time of exit out of Africa [32].

From a phylogeographic perspective, the consistency of the networks across different populations means that all the major haplotypes had originated prior to the differentiation of the study populations. However, the out-of-Africa bottleneck predicts lower diversity levels in non-African compared to African populations, and this is not observed at the *NAT2* locus. There is no marked tendency of any of the derived *NAT2* haplotypes to be continent-specific. In addition, our data display an enhanced frequency of the ancestral *4 haplotype out of Africa, a feature exhibited by only a subset of genes reported in the literature [for reviews see 33], [34].

This indicates that heterogeneity of *NAT2* haplotype frequencies cannot be appropriately predicted by geography alone. Thus, a simple model of variation pre-existent within Africa, spreading out of Africa and evolving by neutral drift is unsatisfactory. Rather, the finding of an independent categorizing variable (subsistence style), which is able to unveil frequency heterogeneity also on a restricted spatial scale [11], [12], and contrasting EHH patterns [8], suggest that *NAT2* variation was shaped by environmental features.

### Deciphering a selective scenario

Several features of the data generated by us and by others [8], [10]–[12] suggest that selection acted on multiple slow-causing variants. There are two main models of selection that may generate patterns of variation similar to those observed at the *NAT2* gene: balancing selection and directional selection on multiple standing variants.

A markedly positive Tajima's D value, as observed at the *NAT2* gene, is expected under balancing selection [35], [36], which can act in the form of heterozygote advantage, frequency-dependent selection or changes in the selective regime over time and/or space. This pattern in the frequency spectrum is a function of the equilibrium frequency of the balanced polymorphism and may be observed in the case of a bi-allelic or a multi-allelic polymorphism [37], [38]. An additional feature of balanced polymorphisms is an excess of diversity, but this is expected only in the case of long-standing selection, i.e. longer than 4N_e_ generations. Therefore, the marked positive Tajima's D value at *NAT2* may be explained by a model of relatively recent balancing selection on *NAT2* non-synonymous variation. The data cannot distinguish between a recent onset and a recent increase in the strength of balancing selection. In either case, the presence of multiple of non-synonymous variants with well documented phenotypic effects suggests selection maintained multiple alleles.

Alternatively, the findings at *NAT2* may be explained by a model of directional selection acting on multiple standing variants, i.e. variants that segregated in the population prior to the onset of selection; these variants may have been completely neutral or slightly deleterious before they became advantageous. Due to the rapid environmental changes occurred during human evolution, a number of investigators have postulated that selection on standing variation (rather than selection on a new beneficial allele) played a major role in human adaptations, thus affording a more rapid adaptive response to the environmental change. A variety of scenarios of directional selection on standing variation have been modelled to determine the expected signature of selection (for reviews see [35], [39], [40]). These models may prove particularly useful to understand the pattern of variation at *NAT2*. Specifically, Pennings and Hermisson [41] showed that Tajima's D may take markedly positive values, as we observe in the *NAT2* coding region, when directional selection acts on multiple existing variants. If multiple variants segregating in a population are concurrently driven to intermediate frequency by positive selection, the variation tightly linked to these variants will also tend to occur at intermediate frequencies, thus generating a skew in the frequency spectrum. In addition, high EHH around the putative advantageous variants may also be observed, though the power of this test for such a selection scenario has not been investigated.

The worldwide pattern of variation at *NAT2* individual SNPs and haplotypes is barely compatible with molecular radiation after population dispersals out of Africa, because it would require later abundant gene flow back into Africa. Conversely, these findings can be easily explained by the presence of *NAT2* polymorphism prior to the exit out of Africa. Within each of the allelic series the repertoire of *NAT2* haplotypes fit the expectations worked out theoretically by Przeworski et al. [42] for a scenario of selection beginning to act on accumulated neutral variation. In this case, many recombination events occur when the allele(s) that will become selected sojourn(s) in the population as neutral variant(s).

However, in one aspect the models proposed by Pennings and Hermisson [41] and Przeworski et al. [42] do not fit the *NAT2* case. In fact they assumed that the beneficial alleles are fixed in the population, thus focusing on a linked neutral locus. Instead, based on biochemical data on the effect of mutations on NAT2 acetylator activity, it is plausible that in the ancestral genetic background, all slow-causing mutations had an equivalent effect on phenotype and fitness. If two or more of these mutations were picked up by selection and simultaneously started to increase in frequency, at some point they started to interfere with each other, thus preventing the fixation of any allele and the sweep itself.

Under such a complex scenario current methods to detect the signature(s) of selection have little power. This may explain the failure of four genome-wide scans in detecting selection at *NAT2*. In the study by Bustamante et al. [43] *NAT2* did not emerge and no genes were reported under positive selection in the GO categories to which it belongs (MF = acyltransferase; BP = other metabolism). No indicator of selection was significant in the study by Voight et al. [44], with peaks of marginal significance only at PSD3, a gene located 200 kb 3′ to *NAT2*. In the analysis by Wang et al. [45], which is based on data largely overlapping with the previous work, *NAT2* falls in a chromosomal region devoid of signals. Finally, no evidence for selection in the *NAT2* region was detected in HapMap phase II data [23].

In conclusion, our data favour the hypothesis that a selective pressure drove at least three slow-causing variants to the frequencies observed today, but do not allow us to discriminate between balancing selection and directional selection on multiple standing variants. While some aspects are in line with balancing selection, parallel directional positive selection favouring all slow causing mutations cannot be ruled out, as was first proposed by Patin et al. [8] when considering the *5 series alone.

### A testable model for a putative selective factor

Our model of selection acting on the *5, *6 and *7 series is supported by the presence of all these haplotypes in widely dispersed populations as contrasted with the strong heterogeneity of their frequencies according to the subsistence style. As populations were sampled in geographically distant locations (see Supplementary Figure S3), co-inheritance of both subsistence style and composition of the gene pool due to shared ancestry is unlikely. Thus, some degree of selection depending on the main food source, or some yet unidentified covariate of it, is the most parsimonious explanation [6 p. 8], [44].

It is to be noted that the two competing selection models mentioned above both imply that the fast-acetylator phenotype began to suffer a selective disadvantage. This is straightforward in the case of positive selection but has to be assumed also in the case of balancing selection, as the frequency of the pooled slow-causing variants is now well above 50% worldwide.

Several studies have pointed out a possible role of NAT enzymes in the catabolism of folates, possibly via the acetylation of p-ABGlu [for reviews see 46], [47]. However, the specific question of the relative roles of enzymes encoded by *NAT1* and *NAT2* in maintaining the overall folate balance in humans has not been directly addressed. Kawamura et al. [3] showed that the affinity of both human NAT1 and NAT2 for p-ABGlu is reduced to a similar extent as compared to each enzyme's best substrate (PABA and hydralazine, respectively). The fact that the liver is the main organ where folate is stored and is also the main site of NAT2 expression, suggests a non negligible role of NAT2 as compared to NAT1.

Different observations indicate possible overlapping effects of folate levels and NATs activity. The overexpression of the orthologue of human *NAT1* in mouse embryos causes developmental abnormalities which are reminiscent of spina bifida-like phenotype [46], [48]. Accordingly, reduced NAT1 activity in humans has been related to a reduced risk of spina bifida [49], a condition well known to be associated with low levels of folates in pregnant mothers.

It is likely that populations shifting from hunting-gathering (H-G) to agriculture became exposed to drastic fluctuations in the supply of folates and to long periods of deprivation. Folates are an essential constituent of the diet, abundant in green leaf vegetables and animal liver, i.e. regular components in the diet of H-G in the terrestrial and marine environment, and in many pastoralist cultures (e.g. the Yakuts). On the contrary, cereals and grains are poor sources of folates, which are further degraded upon storage and cooking [50]. The clinical effects of such a shift, on a short time frame, have been verified in a follow-up study of a !Kung San population [51].

Based on the above elements and the relevance of folates in the etiology of neural tube defects (e.g. OMIM #601634) and in a number of functions related to successful reproduction for reviews see [52], [53], we propose the following model for the increase in frequency of multiple *NAT2* slow haplotypes under changing dietary regimens:

i) A fast acetylating phenotype is neutral (or even advantageous) only in the presence of a folate-rich diet, a situation fulfilled among H-G;
ii) Conversely, when the supply of folates becomes limiting (as might be in the case of a nutritional shift to agriculture), the fitness of fast acetylators drops, due to increased folate loss;
iii) In the same conditions, the slow (or possibly the intermediate) acetylator phenotype, by favouring folate retention, reduce the load caused by fetal loss, birth defects and sub-optimal maternal fertility. Any slow-causing haplotype thus acquires a fitness higher than the fast ones.

The above model relies on the assumption that NAT2 brings an extra contribution to the overall rate of folate catabolism, which is otherwise mainly determined by NAT1. This assumption can be experimentally tested biochemically, *in vitro*, and by correlating NATs phenotypes and folate levels *in vivo*. A necessary corollary is that, while NAT1 activity is strictly physiologically constrained, variation at *NAT2* determines whether the overall rate of folate catabolism becomes critical in the presence of reduced folate supply.

The model easily allows for a small selective advantage of all multiple slow-causing *NAT2* allelic variants, which may explain the growing phase in their frequency trajectory, independently of the underlying biochemical mechanism. The observation that this mechanism often is reduced protein stability [54] leads to the prediction that activity is equally reduced for all substrates, including folate catabolites.

Also, the putative reduction of fitness of fast *NAT2* variants in the presence of low folate supply predicted by our model, applies to a condition that impacts directly on the reproductive success.

On a microgeographic scale, the frequency of *NAT2* slow variants is not easily predictable based on the subsistence style. This is because even populations whose main caloric source are cereals and grains may have historically complemented their diets with valuable sources of folates. This might be the case for Eastern Asian populations, in which the highest frequencies of *4 are observed, despite a rice-based diet (rice being among the poorest sources of folate). For example, in this case, consumption of brown rather than white rice and/or large amounts of fish (as in the Japanese population) may have re-established adequate amounts of folates. Additional aspects to be considered are food diversity, regularity in feeding (hypocaloric stresses being more severe in H-G) and cooking habits (temperature). Finally, further dietary heterogeneity within the three broad categories might be relevant. For example Perry et al. [55] showed that selection pressure on the amylase gene number was related to the dietary input of starch, which is known to vary greatly among H-G's of the arid, rainforest and circum-arctic environments.

In our model, ancestral haplotypes (mainly *4 but also *11, *12 and *13) were compatible with pre-agricultural environmental conditions but begun to determine sub-optimal fitnesses as humans changed lifestyles with or without dispersing into new environmental niches. From this point of view they can be considered *bona fide* ancestral susceptibility alleles [56].

In conclusion, we propose that the present *NAT2* diversity in human populations is the result of three distinct processes: i) presence of variation for slow-causing sites in widely dispersed populations (possibly as neutral variation) before major shifts to pastoralism and/or agriculture as the main modes of food production; ii) independent emergence of selective advantage for multiple slow-causing mutations in populations shifting from H-G to pastoralism/agriculture; iii) further introgression of slow-causing variants into populations anchored to H-G by later gene flow.

## Materials and Methods

### The subjects

Samples from Nigeria (Dendi, coded WAF in Fig. 1), Ethiopia (Amhara [AMH] and Oromo [ORO]), Egypt [EGY], Italy [ITA], Czech Republic [CZK] and Greece [GRE] were previously described [57]–[60], whereas the remaining population samples (i.e. Mordovians [MOE], Perm Russians [PER], Khanti and Mansi [K&M], Yakut [YKL], Chukchee [CHK]) are described here for the first time. All samples were collected personally by one or more of the authors during field campaigns, as consecutive series of unrelated subjects. Field work included the assessment of subsistence style for each population sample. The subjects to be collected were randomly chosen, without selecting for their clinical status or the presence/absence of digestive disturbances.

DNA was extracted from either buccal swab or dried blood adsorbed on paper, with commercially available kits (NucleoSpin, Macherey-Nagel GmbH, Duren, Germany) according to the manufacturer's instructions. Khanty and Mansi subjects were lumped in a single sample. All samples were collected upon written or oral informed consent. This study was approved by the University of Calabria intramural Ethical Committee.

All populations studied here and 35 additional ones from published *NAT2* surveys were classified into the three main subsistence style categories: hunter-gatherers (H-G), pastoralists and agriculturalists as defined in Bromley [61] and references therein or in the original reference (see Supplementary Tables S1 and S6).

### Nomenclature

In contrast with the pharmacogenetic literature, in which the term allele is used to indicate protein variants and corresponding DNA sequences regardless of the number of mutations, we use the term allele to indicate the variant at each nucleotide position and the term haplotype to indicate the overall sequence. Also, note that in the pharmacogenetic literature, protein variants are grouped into series, each of which is characterized by the presence of a specific DNA substitution (for details see http://www.louisville.edu/medschool/pharmacology/NAT.html). We refer to them by omitting the prefix “*NAT2*” (e.g. *4 instead of NAT2*4). Moreover, the pool of series *4, *11, *12 and *13 is referred to as fast acetylator haplotypes, whereas the pool of the remaining series is referred to as slow acetylator haplotypes [7]. Nucleotide positions are numbered according to the coding sequence (Acc. no. D90042.1).

The orthologous *NAT2* sequences for the common chimpanzee (*Pan troglodytes*) and the rhesus macaque (*Macaca mulatta*) were obtained by BLAST using the human coding sequence. These sequences were unambiguously aligned and compared to the human sequence to determine the ancestral state at each polymorphic position.

### Re-sequencing

DNA from all subjects was subjected to a two-step nested PCR protocol to isolate *NAT2* from the paralogous *NAT* genes [8].

Resequencing was performed on the PCR product with standard protocols for ABI310 automatic Sequencer. Electropherograms were aligned with the reference sequence and visually inspected. Ambiguous polymorphisms were confirmed/dismissed by sequencing the opposite strand. The single instance of a novel mutation passing this step, i.e. 34T>C, was further confirmed by restriction analysis as it determines the gain of a *Hae*III site.

### SNP genotyping

The following genotyping protocol was adopted: C282T (rs1041983), C481T (rs1799929), G590A (rs1799930), A803G (rs1208) and G857A (rs1799931) were typed according to a restriction fragment length analysis protocol modified from Deitz et al. [62], on the nested PCR product. T341C (rs1801280) was assayed by an allele-specific PCR protocol modified from Bakayev et al. [63]. Since in compound heterozygotes the products obtained with this allele-specific PCR contain markers in a haploid form, the same assay was also used to determine the phase of specific variants with respect to position 341 by either digestion with restriction enzyme (Supplementary Fig. S1) or re-sequencing.

African samples were also genotyped at the G191A variant (rs1801279), which was previously found only in African populations [8], [14]. This was done by digesting the nested PCR product with *Hap*II in all carriers of haplotypes with the ancestral state at all the positions 341, 590 and 857.

### Data analysis

Haplotypes were inferred in each population sample using the program PHASE (ver. 2) [64]. Genotypes whose phase was reconstructed with posterior probability <0.70, or corresponding to haplotypes never described before (http://www.louisville.edu/medschool/pharmacology/NAT.html), were subjected to experimental validation of the inferred phase, as described above.

The acetylator phenotype for each individual was inferred by assuming that the homozygous or compound heterozygous genotype for two haplotypes of the series *4, *11, *12 or *13 results in the fast acetylator status, the occurrence of one of these haplotypes in combination with a haplotype of the series *5, *6 or *7 results in the intermediate acetylator status and the occurrence of two haplotypes of the series *5, *6 or *7 results in the slow acetylator phenotype [7], [14], [65].

EHH was calculated as described [22] using the program Sweep (http://www.broad.mit.edu/mpg/sweep/index.htm). Core haplotypes were defined for each SNP known to affect NAT2 activity. The EHH for each core haplotype carrying the derived (slow-acetylator) allele was compared to: 1) the EHH for all the remaining haplotypes; and 2) the EHH for the subset of haplotypes carrying the ancestral (fast acetylator) allele at all of the SNPs known to affect NAT2 activity. This analysis was performed on all the data collected in our population samples as well as in the HapMap Phase II data [23] for a region of 100 kb surrounding *NAT2* (chromosome 8 positions 18,250,000 to 18,350,000).

Median joining networks were constructed using the program NETWORK, ver. 4.1.1.2 [66]. The DNAsp package [67] was used to calculate summary statistics of polymorphism data (Theta(pi), Theta(s), haplotype diversity, Tajima's D and Fay & Wu's H) [21], [68], and to perform the McDonald and Kreitman [69] test.

Measures of intra- and inter-population diversity were obtained using the Arlequin 3.01 package [70]. When haplotypes were considered, the fixation indexes were computed with and without information about inter-haplotype molecular distances.

The equality of frequencies across subsistence styles was tested by the non-parametric Kruskal Wallis test as implemented in SPSS.

For all tests of neutrality, coalescent neutral simulations were run under different demographic models to assess the significance of the resequencing data. For all continental samples (i.e. Africa, Europe, Asia), 10,000 replicates were generated using the program MS [71], under three demographic models: i) an exponential growth model with exponential growth rate of 0.183, typical for the African population; ii) bottleneck model A, with a bottleneck of severity 0.1 starting 1200 generations before present and lasting 400 generations, typical for the European population; iii) bottleneck model B, with a bottleneck of severity 0.3 starting 1200 generations before present and lasting 400 generations, typical for the Asian population [72]. The numbers of segregating sites were fixed at the values obtained from the re-sequencing data, the population recombination rate parameter rho ( = 4Nr, where N is the population effective size and r the recombination rate between adjacent sites per generation) was assumed to be 0.24, based on published estimates [73] using the HapMap data for the genomic segment spanning the *NAT2* gene.

## Supporting Information

Figure S1 Typing and phasing of mutations by means of allele-specific PCR. Panel A: PCR specific for alleles at pos. 341. Odd lanes: products specific for allele T. Even lanes: products specific for allele C. Lanes 1–2 and 7–8: results in subjects T/C; lanes 3–4: results in a subject T/T; lanes 5–6: results in a subject C/C. Panel B: Phasing of A803G with respect to T341C in a double heterozygote. PCR products as in panel A (lanes 1–2) were digested with DdeI. Lane 1: Molecular weight marker; lane 2: The product specific for 341(T) [panel A, lane 1] shows the lack of a DdeI site [803(A)], denoted by the 450 bp fragment; lane 3: The product specific for 341(C) [panel A, lane 2] shows the presence of a DdeI site [803(G)], denoted by the 423 bp fragment.
(2.09 MB TIF)

Figure S2 Extended Haplotype Homozygosity (EHH, y axes) vs. physical distance (x axes) for haplotypes carrying mutations which determine the slow acetylator status. The mutations considered are shown on top and are given reference position 0 (vertical bar). For each entry, two plots are shown: in the first one (top) EHH on haplotypes carrying the derived (slow-causing) allele is shown in grey and is compared to EHH on all the remaining haplotypes (in black); in the second one (bottom) the same EHH as above (grey) is compared to the EHH on the subset of fast haplotypes (i.e. carrying the ancestral state at slow-causing positions other than the one assayed) (in black). Note that grey profiles are identical within plot pairs. a) pool of NAT2 haplotypes analysed in the present study (physical distance covering the NAT2 coding region, in bp); b) Yorubans from the HapMap database ; c) Caucasians from the HapMap database; d) Japanese and Chinese from the HapMap database. In b,c,d physical distance covers chromosome 8 positions 18,250,000–18,350,000, in kb from NAT2 5′ end.
(0.25 MB TIF)

Figure S3 Map showing the location of 47 populations for which the frequencies of NAT2 haplotype series have been analysed as a function of subsistence style. Populations are coded as in Supplementary Table S6.
(0.13 MB TIF)

Table S1 (0.05 MB DOC)

Table S2 (1.03 MB DOC)

Table S3 (0.04 MB DOC)

Table S4 (0.04 MB DOC)

Table S5 (0.03 MB DOC)

Table S6 (0.08 MB DOC)
