# Supplementary figures and images for: Multiple Advantageous Amino Acid Variants in the NAT2 Gene in Human Populations
Source: PLoS One. 2008 Sep 5;3(9):e3136. doi: 10.1371/journal.pone.0003136 (PMC2527519; doi:10.1371/journal.pone.0003136)

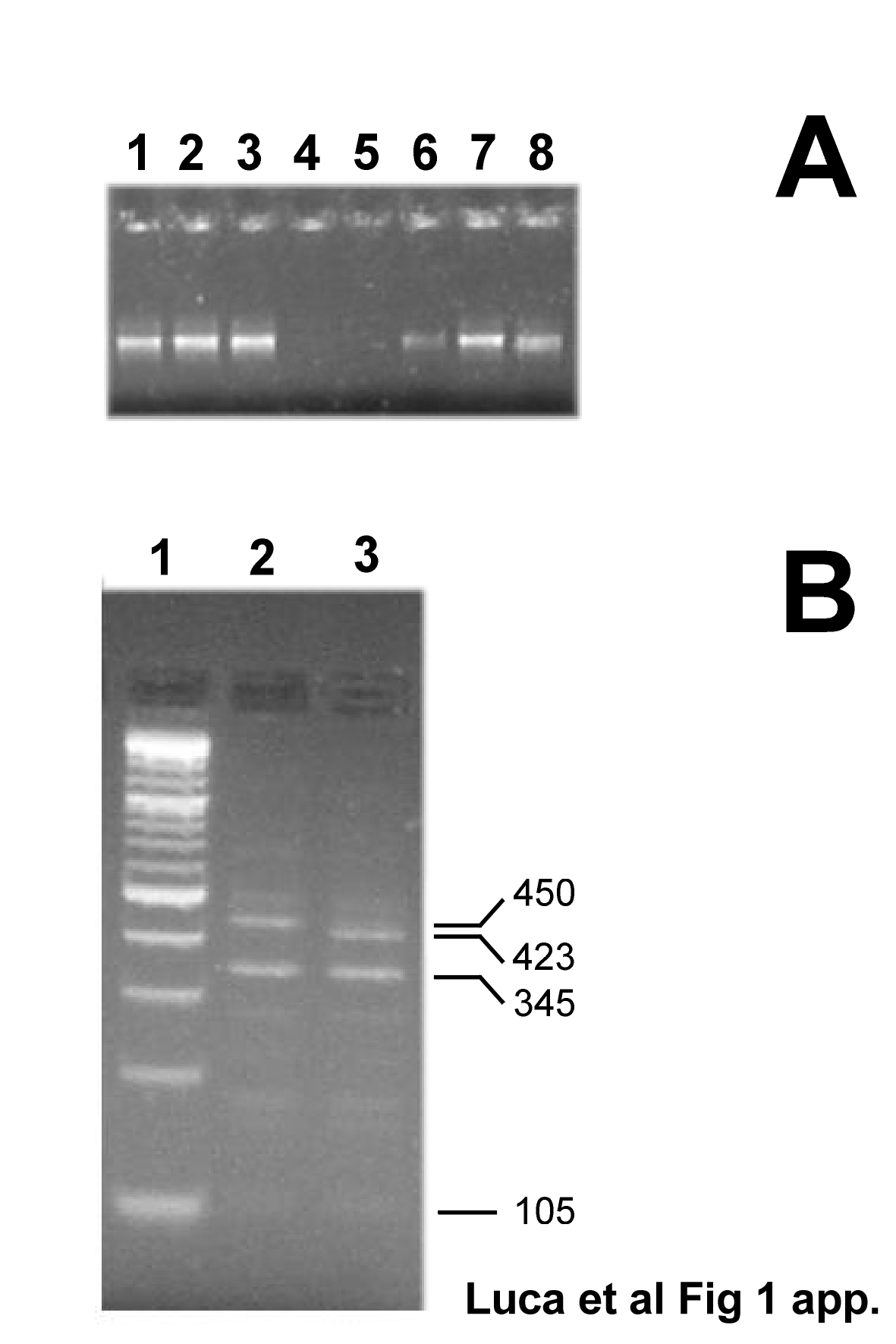

Supplement: Figure S1 — Typing and phasing of mutations by means of allele-specific PCR. Panel A: PCR specific for alleles at pos. 341. Odd lanes: products specific for allele T. Even lanes: products specific for allele C. Lanes 1–2 and 7–8: results in subjects T/C; lanes 3–4: results in a subject T/T; lanes 5–6: results in a subject C/C. Panel B: Phasing of A803G with respect to T341C in a double heterozygote. PCR products as in panel A (lanes 1–2) were digested with DdeI. Lane 1: Molecular weight marker; lane 2: The product specific for 341(T) [panel A, lane 1] shows the lack of a DdeI site [803(A)], denoted by the 450 bp fragment; lane 3: The product specific for 341(C) [panel A, lane 2] shows the presence of a DdeI site [803(G)], denoted by the 423 bp fragment. (2.09 MB TIF) [file pone.0003136.s001.tif]

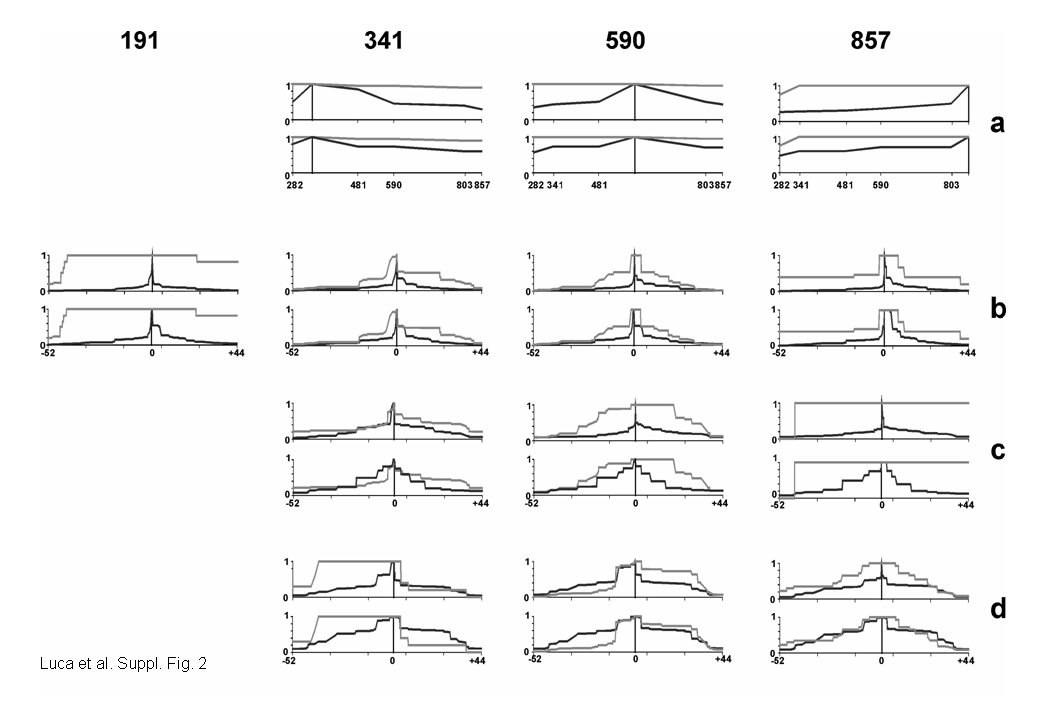

Supplement: Figure S2 — Extended Haplotype Homozygosity (EHH, y axes) vs. physical distance (x axes) for haplotypes carrying mutations which determine the slow acetylator status. The mutations considered are shown on top and are given reference position 0 (vertical bar). For each entry, two plots are shown: in the first one (top) EHH on haplotypes carrying the derived (slow-causing) allele is shown in grey and is compared to EHH on all the remaining haplotypes (in black); in the second one (bottom) the same EHH as above (grey) is compared to the EHH on the subset of fast haplotypes (i.e. carrying the ancestral state at slow-causing positions other than the one assayed) (in black). Note that grey profiles are identical within plot pairs. a) pool of NAT2 haplotypes analysed in the present study (physical distance covering the NAT2 coding region, in bp); b) Yorubans from the HapMap database ; c) Caucasians from the HapMap database; d) Japanese and Chinese from the HapMap database. In b,c,d physical distance covers chromosome 8 positions 18,250,000–18,350,000, in kb from NAT2 5′ end. (0.25 MB TIF) [file pone.0003136.s002.tif]

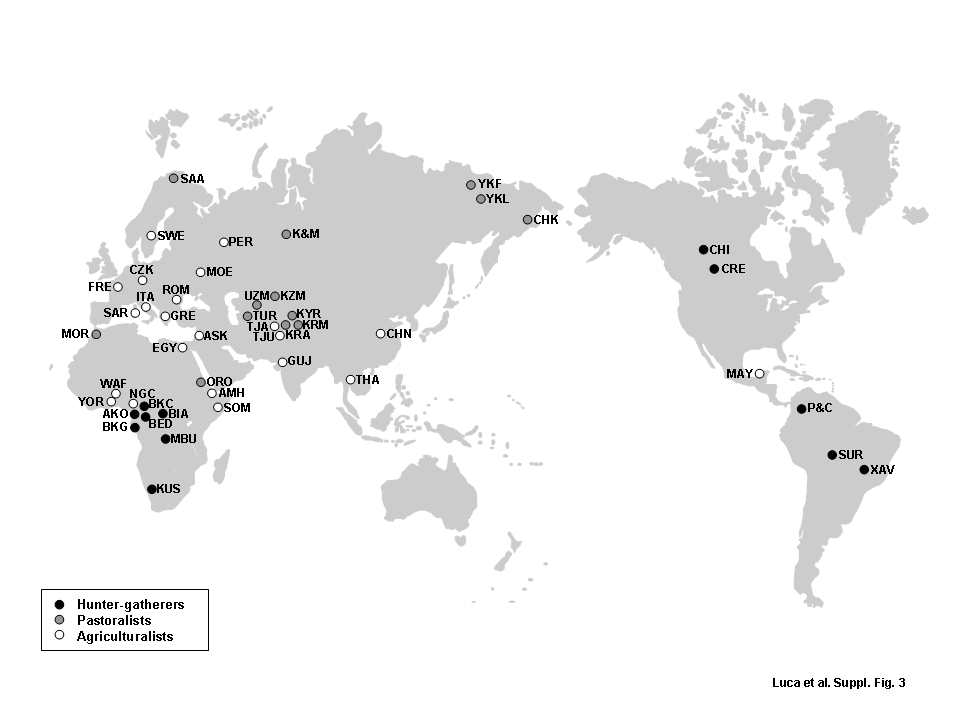

Supplement: Figure S3 — Map showing the location of 47 populations for which the frequencies of NAT2 haplotype series have been analysed as a function of subsistence style. Populations are coded as in Supplementary Table S6. (0.13 MB TIF) [file pone.0003136.s003.tif]
