# Supplementary material for: Multiple Advantageous Amino Acid Variants in the NAT2 Gene in Human Populations
Source: PLoS One. 2008 Sep 5;3(9):e3136. doi: 10.1371/journal.pone.0003136 (PMC2527519; doi:10.1371/journal.pone.0003136)
Supplement: Table S1 — (0.05 MB DOC) [file pone.0003136.s004.doc]

Supplementary Table S1. Absolute frequencies of three NAT2 acetylator phenotypes in the 12 populations here examined. Population codes as in Fig. 1 and Appended Fig. S3.

| Code | Population | Nutritional category | Sequenced | Genotyped | Acetylator status | | | Total |
| --- | --- | --- | --- | --- | --- | --- | --- | --- |
|  |  |  |  |  | Slow | Intermediate | Fast |  |
| WAF | Dendi | Agriculturalist |  | 11 | 8 | 1 | 2 | 11 |
| AMH | Amhara | Agriculturalist | 6 | 9 | 11 | 4 | 0 | 15 |
| ORO | Oromo | Pastoralist |  | 12 | 7 | 5 | 0 | 12 |
| EGY | Egyptians | Agriculturalist | 10 | 27 | 25 | 9 | 3 | 37 |
| ITA | Italians | Agriculturalist | 8 | 29 | 18 | 17 | 2 | 37 |
| GRE | Greeks | Agriculturalist | 10 | 30 | 23 | 15 | 2 | 40 |
| CZK | Czechs | Agriculturalist |  | 27 | 17 | 9 | 1 | 27 |
| MOE | Mordvin | Agriculturalist | 14 |  | 7 | 5 | 2 | 14 |
| PER | Russian | Agriculturalist | 12 |  | 4 | 4 | 4 | 12 |
| K&M | Khanty&Mansi | Pastoralist | 14 |  | 6 | 6 | 2 | 14 |
| YKL | Yakuts | Pastoralist | 11 | 5 | 6 | 7 | 3 | 16 |
| CHK | Chukchee | Pastoralist | 13 |  | 6 | 4 | 3 | 13 |
|  |  |  |  |  |  |  |  |  |
|  | TOTAL |  | 98 | 150 | 138 | 86 | 24 | 248 |
