# Supplementary material for: Multiple Advantageous Amino Acid Variants in the NAT2 Gene in Human Populations
Source: PLoS One. 2008 Sep 5;3(9):e3136. doi: 10.1371/journal.pone.0003136 (PMC2527519; doi:10.1371/journal.pone.0003136)
Supplement: Table S2 — (1.03 MB DOC) [file pone.0003136.s005.doc]

Supplementary Table S2. Sequencing (S) and genotyping (G) results in the 248 subjects here examined. Each line represents the allele state at each polymorphic position resulting from the phasing procedures described in Materials and Methods.

|  | Pop.  code | **Subject** | **282** | **341** | **481** | **590** | **803** | **857** | **Haplotype** | **Acetylator Status** |
| --- | --- | --- | --- | --- | --- | --- | --- | --- | --- | --- |
| S | CHK | CHK007 | T | T | C | A | A | G | NAT2*6A | Slow |
| S | CHK |  | T | T | C | A | A | G | NAT2*6A |  |
| S | CHK | CHK023 | C | T | C | G | A | G | NAT2*4 | Fast |
| S | CHK |  | C | T | C | G | A | G | NAT2*4 |  |
| S | CHK | CHK080 | T | T | C | A | G | G | NAT2*6C | Slow |
| S | CHK |  | C | C | T | G | G | G | NAT2*5B |  |
| S | CHK | CHK082 | C | T | C | G | A | G | NAT2*4 | Fast |
| S | CHK |  | C | T | C | G | A | G | NAT2*4 |  |
| S | CHK | CHK088 | T | T | C | A | A | G | NAT2*6A | Slow |
| S | CHK |  | T | T | C | A | A | G | NAT2*6A |  |
| S | CHK | CHK092 | C | T | C | G | G | G | NAT2*12A | Intermediate |
| S | CHK |  | C | C | T | G | G | G | NAT2*5B |  |
| S | CHK | CHK155 | T | T | C | A | A | G | NAT2*6A | Slow |
| S | CHK |  | T | T | C | A | A | G | NAT2*6A |  |
| S | CHK | CHK161 | C | C | T | G | G | G | NAT2*5B | Slow |
| S | CHK |  | C | C | T | G | G | G | NAT2*5B |  |
| S | CHK | CHK163 | T | T | C | G | A | A | NAT2*7B | Slow |
| S | CHK |  | T | T | C | G | A | A | NAT2*7B |  |
| S | CHK | CHK164 | C | T | C | G | A | G | NAT2*4 | Fast |
| S | CHK |  | C | T | C | G | A | G | NAT2*4 |  |
| S | CHK | CHK167 | C | T | C | G | A | G | NAT2*4 | Intermediate |
| S | CHK |  | C | T | C | G | A | A | NAT2*7A |  |
| S | CHK | CHK174 | C | T | C | G | A | G | NAT2*4 | Intermediate |
| S | CHK |  | C | C | T | G | G | G | NAT2*5B |  |
| S | CHK | CHK177 | T | T | C | A | A | G | NAT2*6A | Intermediate |
| S | CHK |  | C | T | C | G | A | G | NAT2*4 |  |
| G | CZK | CK102 | C | C | C | G | A | G | NAT2*5D | Slow |
| G | CZK |  | C | C | T | G | G | G | NAT2*5B |  |
| G | CZK | CK103 | C | T | C | G | A | G | NAT2*4 | Intermediate |
| G | CZK |  | T | T | C | A | A | G | NAT2*6A |  |
| G | CZK | CK114 | C | C | T | G | G | G | NAT2*5B | Slow |
| G | CZK |  | T | T | C | A | A | G | NAT2*6A |  |
| G | CZK | CK129 | C | C | T | G | G | G | NAT2*5B | Slow |
| G | CZK |  | T | T | C | A | A | G | NAT2*6A |  |
| G | CZK | CK201 | C | C | C | G | A | G | NAT2*5D | Slow |
| G | CZK |  | C | C | T | G | G | G | NAT2*5B |  |
| G | CZK | CK204 | C | T | C | G | A | G | NAT2*4 | Fast |
| G | CZK |  | C | T | C | G | A | G | NAT2*4 |  |
| G | CZK | CK233 | C | C | T | G | G | G | NAT2*5B | Intermediate |
| G | CZK |  | C | T | C | G | A | G | NAT2*4 |  |
| G | CZK | CK234 | C | C | T | G | G | G | NAT2*5B | Slow |
| G | CZK |  | C | C | T | G | G | G | NAT2*5B |  |
| G | CZK | CK240 | C | C | T | G | G | G | NAT2*5B | Intermediate |
| G | CZK |  | C | T | C | G | A | G | NAT2*4 |  |
| G | CZK | CK301 | C | C | C | G | G | G | NAT2*5C | Slow |
| G | CZK |  | T | T | C | A | A | G | NAT2*6A |  |
| G | CZK | CK304 | C | C | C | G | G | G | NAT2*5C | Slow |
| G | CZK |  | C | C | T | G | G | G | NAT2*5B |  |
| G | CZK | CK311 | C | C | T | G | G | G | NAT2*5B | Slow |
| G | CZK |  | C | C | T | G | G | G | NAT2*5B |  |
| G | CZK | CK315 | C | C | T | G | G | G | NAT2*5B | Slow |
| G | CZK |  | T | C | C | A | A | G | NAT2*5J |  |
| G | CZK | CK316 | C | T | C | G | A | G | NAT2*4 | Intermediate |
| G | CZK |  | T | T | C | A | A | G | NAT2*6A |  |
| G | CZK | CK329 | C | C | T | G | G | G | NAT2*5B | Intermediate |
| G | CZK |  | C | T | C | G | A | G | NAT2*4 |  |
| G | CZK | CK338 | C | C | C | G | G | G | NAT2*5C | Intermediate |
| G | CZK |  | C | T | C | G | A | G | NAT2*4 |  |
| G | CZK | CK347 | T | T | C | A | A | G | NAT2*6A | Slow |
| G | CZK |  | T | T | C | A | A | G | NAT2*6A |  |
| G | CZK | CK348 | C | C | T | G | G | G | NAT2*5B | Slow |
| G | CZK |  | C | C | T | G | G | G | NAT2*5B |  |
| G | CZK | CK352 | C | T | C | G | A | G | NAT2*4 | Intermediate |
| G | CZK |  | T | T | C | A | A | G | NAT2*6A |  |
| G | CZK | CK356 | C | C | T | G | G | G | NAT2*5B | Slow |
| G | CZK |  | T | T | C | A | A | G | NAT2*6A |  |
| G | CZK | CK364 | C | C | T | G | G | G | NAT2*5B | Slow |
| G | CZK |  | T | T | C | A | A | G | NAT2*6A |  |
| G | CZK | CK414 | C | C | T | G | G | G | NAT2*5B | Slow |
| G | CZK |  | T | C | C | A | A | G | NAT2*5J |  |
| G | CZK | CK424 | C | T | C | G | A | G | NAT2*4 | Intermediate |
| G | CZK |  | T | T | C | A | A | G | NAT2*6A |  |
| G | CZK | CK431 | C | C | T | G | G | G | NAT2*5B | Slow |
| G | CZK |  | T | T | C | A | A | G | NAT2*6A |  |
| G | CZK | CK433 | C | C | C | G | A | G | NAT2*5D | Slow |
| G | CZK |  | C | C | T | G | G | G | NAT2*5B |  |
| G | CZK | CK439 | T | T | C | A | A | G | NAT2*6A | Slow |
| G | CZK |  | T | T | C | A | A | G | NAT2*6A |  |
| G | CZK | CK440 | C | C | T | G | G | G | NAT2*5B | Intermediate |
| G | CZK |  | C | T | C | G | A | G | NAT2*4 |  |
| S | K&M | CM149 | C | C | T | G | G | G | NAT2*5B | Slow |
| S | K&M |  | C | C | T | G | G | G | NAT2*5B |  |
| S | K&M | CM150 | C | C | C | G | G | G | NAT2*5C | Intermediate |
| S | K&M |  | C | T | C | G | G | G | NAT2*12A |  |
| S | K&M | CM151 | C | C | T | G | G | G | NAT2*5B | Intermediate |
| S | K&M |  | C | T | C | G | A | G | NAT2*4 |  |
| S | K&M | CM153 | C | C | T | G | G | G | NAT2*5B | Slow |
| S | K&M |  | T | T | C | A | A | G | NAT2*6A |  |
| S | K&M | CM162 | C | C | T | G | G | G | NAT2*5B | Slow |
| S | K&M |  | T | T | C | G | A | A | NAT2*7B |  |
| S | K&M | CM170 | C | C | T | G | G | G | NAT2*5B | Slow |
| S | K&M |  | C | C | T | G | G | G | NAT2*5B |  |
| S | K&M | CM180 | C | T | C | G | A | G | NAT2*4 | Intermediate |
| S | K&M |  | T | T | C | G | A | A | NAT2*7B |  |
| S | K&M | CM35 | C | T | T | G | A | G | NAT2*11A | Intermediate |
| S | K&M |  | T | T | C | G | A | A | NAT2*7B |  |
| S | K&M | CM36 | C | T | C | G | A | G | NAT2*4 | Fast |
| S | K&M |  | C | T | C | G | A | G | NAT2*4 |  |
| S | K&M | CM56 | T | T | C | A | A | G | NAT2*6A | Slow |
| S | K&M |  | T | T | C | A | A | G | NAT2*6A |  |
| S | K&M | MM152 | C | C | T | G | G | G | NAT2*5B | Slow |
| S | K&M |  | C | C | C | G | G | G | NAT2*5C |  |
| S | K&M | MM156 | C | T | T | G | A | G | NAT2*11A | Fast |
| S | K&M |  | C | T | T | G | A | G | NAT2*11A |  |
| S | K&M | MM171 | C | T | T | G | G | G | NAT2*12C | Intermediate |
| S | K&M |  | T | T | C | G | A | A | NAT2*7B |  |
| S | K&M | MM87 | T | T | C | G | A | G | NAT2*13 | Intermediate |
| S | K&M |  | T | T | C | G | A | A | NAT2*7B |  |
| G | EGY | 16cf3 | C | T | C | G | A | G | NAT2*4 | Intermediate |
| G | EGY |  | C | C | T | G | G | G | NAT2*5B |  |
| G | EGY | E64cf1 | C | C | T | G | G | G | NAT2*5B | Slow |
| G | EGY |  | C | C | T | G | G | G | NAT2*5B |  |
| G | EGY | E315 | C | T | C | G | A | G | NAT2*4 | Intermediate |
| G | EGY |  | T | T | C | G | A | A | NAT2*7B |  |
| G | EGY | E404 | C | T | C | G | G | G | NAT2*12A | Intermediate |
| G | EGY |  | C | C | T | G | G | G | NAT2*5B |  |
| G | EGY | AL4 | C | C | T | G | G | G | NAT2*5B | Slow |
| G | EGY |  | T | T | C | A | A | G | NAT2*6A |  |
| G | EGY | E18cf3 | C | C | T | G | G | G | NAT2*5B | Slow |
| G | EGY |  | C | C | T | G | G | G | NAT2*5B |  |
| G | EGY | E1cf3 | C | C | C | G | G | G | NAT2*5C | Slow |
| G | EGY |  | C | C | T | G | G | G | NAT2*5B |  |
| G | EGY | E24cf3 | C | C | T | G | G | G | NAT2*5B | Slow |
| G | EGY |  | T | T | C | A | A | G | NAT2*6A |  |
| G | EGY | E263 | C | C | T | G | G | G | NAT2*5B | Slow |
| G | EGY |  | T | T | C | A | A | G | NAT2*6A |  |
| G | EGY | E504 | C | C | T | G | G | G | NAT2*5B | Slow |
| G | EGY |  | T | T | C | A | A | G | NAT2*6A |  |
| G | EGY | 520 | C | T | C | G | A | G | NAT2*4 | Intermediate |
| G | EGY |  | T | T | C | A | A | G | NAT2*6A |  |
| G | EGY | E332 | C | C | T | G | G | G | NAT2*5B | Slow |
| G | EGY |  | T | T | C | A | A | G | NAT2*6A |  |
| G | EGY | E138 | C | T | C | G | A | G | NAT2*4 | Fast |
| G | EGY |  | T | T | C | G | A | G | NAT2*13 |  |
| G | EGY | EG418 | C | C | T | G | G | G | NAT2*5B | Intermediate |
| G | EGY |  | T | T | C | G | A | G | NAT2*13 |  |
| G | EGY | E87 | C | C | T | G | G | G | NAT2*5B | Slow |
| G | EGY |  | C | C | T | G | G | G | NAT2*5B |  |
| G | EGY | E284 | C | T | C | G | A | G | NAT2*4 | Intermediate |
| G | EGY |  | C | C | T | G | A | G | NAT2*5A |  |
| G | EGY | E30cf3 | C | C | T | G | G | G | NAT2*5B | Slow |
| G | EGY |  | T | T | C | A | A | G | NAT2*6A |  |
| G | EGY | E164 | C | C | T | G | G | G | NAT2*5B | Slow |
| G | EGY |  | T | T | C | A | A | G | NAT2*6A |  |
| G | EGY | E459 | C | C | T | G | G | G | NAT2*5B | Intermediate |
| G | EGY |  | T | T | C | G | A | G | NAT2*13 |  |
| G | EGY | E448 | T | T | C | A | A | G | NAT2*6A | Slow |
| G | EGY |  | T | T | C | A | A | G | NAT2*6A |  |
| G | EGY | E90 | C | C | T | G | G | G | NAT2*5B | Slow |
| G | EGY |  | T | T | C | A | A | G | NAT2*6A |  |
| G | EGY | E31cf1 | C | C | T | G | G | G | NAT2*5B | Slow |
| G | EGY |  | C | C | T | G | G | G | NAT2*5B |  |
| G | EGY | E50cf1 | C | C | T | G | G | G | NAT2*5B | Slow |
| G | EGY |  | C | C | T | G | G | G | NAT2*5B |  |
| G | EGY | E35cf3 | C | C | T | G | G | G | NAT2*5B | Slow |
| G | EGY |  | C | C | T | G | G | G | NAT2*5B |  |
| G | EGY | E312 | T | T | C | A | A | G | NAT2*6A | Slow |
| G | EGY |  | T | T | C | A | A | G | NAT2*6A |  |
| G | EGY | E317 | T | T | C | A | A | G | NAT2*6A | Slow |
| G | EGY |  | T | T | C | A | A | G | NAT2*6A |  |
| G | EGY | E539 | T | T | C | G | A | G | NAT2*13 | Intermediate |
| G | EGY |  | T | T | C | A | A | G | NAT2*6A |  |
| S | EGY | AL36 | C | C | T | G | G | G | NAT2*5B | Slow |
| S | EGY |  | C | C | T | G | G | G | NAT2*5B |  |
| S | EGY | AL37 | C | C | T | G | G | G | NAT2*5B | Slow |
| S | EGY |  | C | C | T | G | G | G | NAT2*5B |  |
| S | EGY | AL39 | C | T | C | G | A | G | NAT2*4 | Fast |
| S | EGY |  | C | T | C | G | A | G | NAT2*4 |  |
| S | EGY | AL40 | C | T | C | G | A | G | NAT2*4 | Fast |
| S | EGY |  | T | T | C | G | A | G | NAT2*13 |  |
| S | EGY | AL41 | T | T | C | A | A | G | NAT2*6A | Slow |
| S | EGY |  | T | T | C | A | A | G | NAT2*6A |  |
| S | EGY | AL42 | T | T | C | A | A | G | NAT2*6A | Slow |
| S | EGY |  | T | T | C | A | A | G | NAT2*6A |  |
| S | EGY | AL44 | C | C | T | G | G | G | NAT2*5B | Slow |
| S | EGY |  | C | C | T | G | G | G | NAT2*5B |  |
| S | EGY | AL45 | C | T | C | G | A | G | NAT2*4 | Intermediate |
| S | EGY |  | C | C | T | G | G | G | NAT2*5B |  |
| S | EGY | AL46 | C | C | T | G | G | G | NAT2*5B | Slow |
| S | EGY |  | C | C | T | G | G | G | NAT2*5B |  |
| S | EGY | AL51 | C | C | T | G | G | G | NAT2*5B | Slow |
| S | EGY |  | C | C | T | G | G | G | NAT2*5B |  |
| S | AMH | AMH002 | C | C | T | G | G | G | NAT2*5B | Slow |
| S | AMH |  | C | C | T | G | G | G | NAT2*5B |  |
| S | AMH | AMH133 | C | C | T | G | G | G | NAT2*5B | Slow |
| S | AMH |  | C | C | T | G | G | G | NAT2*5B |  |
| S | AMH | AMH166 | C | C | T | G | G | G | NAT2*5B | Slow |
| S | AMH |  | T | T | C | A | A | G | NAT2*6A |  |
| S | AMH | AMH168 | C | C | T | G | G | G | NAT2*5B | Slow |
| S | AMH |  | C | C | T | G | G | G | NAT2*5B |  |
| S | AMH | AMH174 | C | C | T | G | G | G | NAT2*5B | Slow |
| S | AMH |  | T | T | C | A | A | G | NAT2*6A |  |
| S | AMH | AMH178 | C | C | T | G | G | G | NAT2*5B | Slow |
| S | AMH |  | T | T | C | A | A | G | NAT2*6A |  |
| G | AMH | AMH27 | C | T | C | G | G | G | NAT2*12A | Intermediate |
| G | AMH |  | T | T | C | A | A | G | NAT2*6A |  |
| G | AMH | AMH170 | C | C | T | G | G | G | NAT2*5B | Slow |
| G | AMH |  | C | C | T | G | G | G | NAT2*5B |  |
| G | AMH | AMH150 | T | T | C | G | A | A | NAT2*7B | Slow |
| G | AMH |  | T | T | C | A | A | G | NAT2*6A |  |
| G | AMH | AMH141 | C | C | T | G | G | G | NAT2*5B | Slow |
| G | AMH |  | C | C | T | G | G | G | NAT2*5B |  |
| G | AMH | AMH24 | C | T | C | G | A | G | NAT2*4 | Intermediate |
| G | AMH |  | T | T | C | A | A | G | NAT2*6A |  |
| G | AMH | AMH151 | C | C | T | G | G | G | NAT2*5B | Slow |
| G | AMH |  | T | T | C | A | A | G | NAT2*6A |  |
| G | AMH | AMH172 | C | C | T | G | G | G | NAT2*5B | Intermediate |
| G | AMH |  | C | T | C | G | A | G | NAT2*4 |  |
| G | AMH | AMH157 | C | C | T | G | G | G | NAT2*5B | Slow |
| G | AMH |  | T | T | C | A | A | G | NAT2*6A |  |
| G | AMH | AMH167 | T | C | T | G | G | G | NAT2*5G | Intermediate |
| G | AMH |  | T | T | C | G | A | G | NAT2*13 |  |
| G | ORO | ORO30 | C | C | T | G | G | G | NAT2*5B | Slow |
| G | ORO |  | C | C | T | G | G | G | NAT2*5B |  |
| G | ORO | ORO39 | C | C | T | G | G | G | NAT2*5B | Slow |
| G | ORO |  | C | C | T | G | A | G | NAT2*5A |  |
| G | ORO | ORO54 | C | C | T | G | G | G | NAT2*5B | Slow |
| G | ORO |  | T | T | C | G | A | A | NAT2*7B |  |
| G | ORO | ORO86 | C | C | T | G | G | G | NAT2*5B | Slow |
| G | ORO |  | T | T | C | A | A | G | NAT2*6A |  |
| G | ORO | ORO87 | T | T | C | G | A | G | NAT2*13 | Intermediate |
| G | ORO |  | T | T | C | A | A | G | NAT2*6A |  |
| G | ORO | ORO107 | C | T | C | G | A | G | NAT2*4 | Intermediate |
| G | ORO |  | T | T | C | G | A | A | NAT2*7B |  |
| G | ORO | ORO126 | C | C | T | G | G | G | NAT2*5B | Intermediate |
| G | ORO |  | C | T | C | G | G | G | NAT2*12A |  |
| G | ORO | ORO128 | C | C | T | G | G | G | NAT2*5B | Intermediate |
| G | ORO |  | C | T | C | G | G | G | NAT2*12A |  |
| G | ORO | ORO140 | C | C | T | G | G | G | NAT2*5B | Slow |
| G | ORO |  | T | T | C | A | A | G | NAT2*6A |  |
| G | ORO | ORO171 | C | C | C | G | G | G | NAT2*5C | Slow |
| G | ORO |  | T | T | C | A | A | G | NAT2*6A |  |
| G | ORO | ORO191 | T | T | C | G | A | A | NAT2*7B | Slow |
| G | ORO |  | T | T | C | A | A | G | NAT2*6A |  |
| G | ORO | ORO189 | C | T | C | G | A | G | NAT2*4 | Intermediate |
| G | ORO |  | T | T | C | G | A | A | NAT2*7B |  |
| G | GRE | GR99-02 | C | C | T | G | G | G | NAT2*5B | Slow |
| G | GRE |  | T | T | C | A | A | G | NAT2*6A |  |
| G | GRE | GR99-03 | C | C | T | G | G | G | NAT2*5B | Slow |
| G | GRE |  | T | T | C | A | A | G | NAT2*6A |  |
| G | GRE | GR99-06 | C | T | C | G | A | G | NAT2*4 | Intermediate |
| G | GRE |  | C | C | T | G | G | G | NAT2*5B |  |
| G | GRE | GR99-11 | C | T | C | G | A | G | NAT2*4 | Intermediate |
| G | GRE |  | C | C | C | G | G | G | NAT2*5C |  |
| G | GRE | GR99-18 | T | T | C | A | A | G | NAT2*6A | Slow |
| G | GRE |  | T | T | C | G | A | A | NAT2*7B |  |
| G | GRE | GR99-25 | C | C | T | G | G | G | NAT2*5B | Slow |
| G | GRE |  | C | C | T | G | G | G | NAT2*5B |  |
| G | GRE | GR99-26 | C | T | C | G | A | G | NAT2*4 | Intermediate |
| G | GRE |  | T | T | C | A | A | G | NAT2*6A |  |
| G | GRE | GR99-37 | C | C | T | G | G | G | NAT2*5B | Slow |
| G | GRE |  | T | T | C | A | A | G | NAT2*6A |  |
| G | GRE | GR99-43 | C | T | C | G | A | G | NAT2*4 | Intermediate |
| G | GRE |  | C | C | T | G | G | G | NAT2*5B |  |
| G | GRE | GR99-77 | C | C | T | G | G | G | NAT2*5B | Slow |
| G | GRE |  | C | C | T | G | G | G | NAT2*5B |  |
| G | GRE | GR99-79 | C | T | C | G | A | G | NAT2*4 | Intermediate |
| G | GRE |  | T | T | C | A | A | G | NAT2*6A |  |
| G | GRE | GR99-85 | C | T | C | A | A | G | NAT2*6B | Slow |
| G | GRE |  | T | T | C | A | A | G | NAT2*6A |  |
| G | GRE | GR99-101 | C | T | C | G | A | G | NAT2*4 | Intermediate |
| G | GRE |  | C | C | C | G | G | G | NAT2*5C |  |
| G | GRE | GR99-103 | C | T | C | G | A | G | NAT2*4 | Intermediate |
| G | GRE |  | C | C | T | G | G | G | NAT2*5B |  |
| G | GRE | GR99-108 | C | T | C | G | A | G | NAT2*4 | Intermediate |
| G | GRE |  | T | T | C | A | A | G | NAT2*6A |  |
| G | GRE | GR99-110 | C | C | T | G | G | G | NAT2*5B | Slow |
| G | GRE |  | T | T | C | A | A | G | NAT2*6A |  |
| G | GRE | GR99-116 | C | C | T | G | G | G | NAT2*5B | Slow |
| G | GRE |  | T | T | C | A | A | G | NAT2*6A |  |
| G | GRE | GR99-120 | C | C | T | G | G | G | NAT2*5B | Slow |
| G | GRE |  | C | C | T | G | G | G | NAT2*5B |  |
| G | GRE | GR99-126 | C | C | C | G | G | G | NAT2*5C | Slow |
| G | GRE |  | C | C | T | G | G | G | NAT2*5B |  |
| G | GRE | GR99-139 | C | C | T | G | G | G | NAT2*5B | Slow |
| G | GRE |  | T | T | C | A | A | G | NAT2*6A |  |
| G | GRE | GR99-151 | C | T | C | G | A | G | NAT2*4 | Intermediate |
| G | GRE |  | T | T | C | A | A | G | NAT2*6A |  |
| G | GRE | GR99-161 | T | T | C | A | A | G | NAT2*6A | Slow |
| G | GRE |  | T | T | C | A | A | G | NAT2*6A |  |
| G | GRE | GR99-172 | C | T | C | G | A | G | NAT2*4 | Fast |
| G | GRE |  | C | T | C | G | A | G | NAT2*4 |  |
| G | GRE | GR99-174 | C | C | T | G | G | G | NAT2*5B | Slow |
| G | GRE |  | T | T | C | A | A | G | NAT2*6A |  |
| G | GRE | GR99-178 | T | T | C | A | A | G | NAT2*6A | Slow |
| G | GRE |  | T | T | C | A | A | G | NAT2*6A |  |
| G | GRE | GR99-179 | C | T | C | G | A | G | NAT2*4 | Intermediate |
| G | GRE |  | C | C | T | G | G | G | NAT2*5B |  |
| G | GRE | GR99-186 | C | C | T | G | G | G | NAT2*5B | Slow |
| G | GRE |  | T | T | C | A | A | G | NAT2*6A |  |
| G | GRE | GR99-188 | C | C | T | G | G | G | NAT2*5B | Slow |
| G | GRE |  | C | C | T | G | G | G | NAT2*5B |  |
| G | GRE | GR99-195 | C | T | C | G | A | G | NAT2*4 | Fast |
| G | GRE |  | C | T | C | G | A | G | NAT2*4 |  |
| G | GRE | GR99-202 | C | C | T | G | A | G | NAT2*5A | Slow |
| G | GRE |  | C | C | T | G | G | G | NAT2*5B |  |
| S | GRE | GR0401 | C | C | T | G | G | G | NAT2*5B | Slow |
| S | GRE |  | T | T | C | A | A | G | NAT2*6A |  |
| S | GRE | GR0402 | C | C | T | G | G | G | NAT2*5B | Slow |
| S | GRE |  | T | T | C | G | A | A | NAT2*7B |  |
| S | GRE | GR0403 | C | T | C | G | A | G | NAT2*4 | Intermediate |
| S | GRE |  | T | T | C | A | A | G | NAT2*6A |  |
| S | GRE | GR0404 | C | C | T | G | G | G | NAT2*5B | Slow |
| S | GRE |  | T | T | C | A | A | G | NAT2*6A |  |
| S | GRE | GR0405 | C | C | T | G | G | G | NAT2*5B | Slow |
| S | GRE |  | T | T | C | A | A | G | NAT2*6A |  |
| S | GRE | GR0406 | C | T | C | G | A | G | NAT2*4 | Intermediate |
| S | GRE |  | C | T | C | G | A | A | NAT2*7A |  |
| S | GRE | GR0407 | C | C | T | G | G | G | NAT2*5B | Slow |
| S | GRE |  | T | T | C | A | A | G | NAT2*6A |  |
| S | GRE | GR0408 | C | T | T | G | G | G | NAT2*12C | Intermediate |
| S | GRE |  | C | C | T | G | G | G | NAT2*5B |  |
| S | GRE | GR0409 | C | T | C | G | A | G | NAT2*4 | Intermediate |
| S | GRE |  | C | C | T | G | G | G | NAT2*5B |  |
| S | GRE | GR0410 | C | C | T | G | G | G | NAT2*5B | Intermediate |
| S | GRE |  | T | T | C | G | A | G | NAT2*13 |  |
| G | ITA | PAL11 | C | T | C | G | A | G | NAT2*4 | Intermediate |
| G | ITA |  | C | C | T | G | G | G | NAT2*5B |  |
| G | ITA | PAL12 | C | T | C | G | A | G | NAT2*4 | Intermediate |
| G | ITA |  | C | C | T | G | G | G | NAT2*5B |  |
| G | ITA | PAL13 | C | T | C | G | A | G | NAT2*4 | Intermediate |
| G | ITA |  | T | T | C | A | A | G | NAT2*6A |  |
| G | ITA | PAL14 | C | C | T | G | G | G | NAT2*5B | Slow |
| G | ITA |  | T | T | C | A | A | G | NAT2*6A |  |
| G | ITA | PAL15 | C | T | C | G | A | G | NAT2*4 | Intermediate |
| G | ITA |  | T | T | C | A | A | G | NAT2*6A |  |
| G | ITA | PAL16 | C | T | C | G | A | G | NAT2*4 | Fast |
| G | ITA |  | C | T | C | G | A | G | NAT2*4 |  |
| G | ITA | PAL17 | C | T | C | G | A | G | NAT2*4 | Intermediate |
| G | ITA |  | T | T | C | A | A | G | NAT2*6A |  |
| G | ITA | PAL18 | C | C | T | G | G | G | NAT2*5B | Slow |
| G | ITA |  | T | T | C | A | A | G | NAT2*6A |  |
| G | ITA | PAL19 | C | C | T | G | G | G | NAT2*5B | Slow |
| G | ITA |  | T | T | C | A | A | G | NAT2*6A |  |
| G | ITA | PAL20 | C | T | C | G | A | G | NAT2*4 | Intermediate |
| G | ITA |  | C | C | T | G | G | G | NAT2*5B |  |
| G | ITA | PAL21 | C | T | C | G | A | G | NAT2*4 | Intermediate |
| G | ITA |  | T | T | C | G | A | A | NAT2*7B |  |
| G | ITA | PAL23 | C | C | T | G | G | G | NAT2*5B | Slow |
| G | ITA |  | T | T | C | G | A | A | NAT2*7B |  |
| G | ITA | PAL24 | C | C | T | G | G | G | NAT2*5B | Slow |
| G | ITA |  | C | C | C | G | G | G | NAT2*5C |  |
| G | ITA | PAL25 | C | C | T | G | G | G | NAT2*5B | Slow |
| G | ITA |  | T | T | C | A | A | G | NAT2*6A |  |
| G | ITA | PAL26 | C | T | C | G | A | G | NAT2*4 | Intermediate |
| G | ITA |  | T | T | C | A | A | G | NAT2*6A |  |
| G | ITA | PAL27 | C | T | C | G | A | G | NAT2*4 | Intermediate |
| G | ITA |  | T | T | C | A | A | G | NAT2*6A |  |
| G | ITA | PAL28 | C | C | T | G | G | G | NAT2*5B | Slow |
| G | ITA |  | T | T | C | A | A | G | NAT2*6A |  |
| G | ITA | PAL29 | C | C | T | G | G | G | NAT2*5B | Slow |
| G | ITA |  | T | T | C | A | A | G | NAT2*6A |  |
| G | ITA | PAL30 | C | C | C | G | G | G | NAT2*5C | Slow |
| G | ITA |  | T | T | C | A | A | G | NAT2*6A |  |
| G | ITA | PAL31 | C | C | T | G | G | G | NAT2*5B | Slow |
| G | ITA |  | T | T | C | A | A | G | NAT2*6A |  |
| G | ITA | PAL32 | C | T | C | G | A | G | NAT2*4 | Intermediate |
| G | ITA |  | C | C | T | G | G | G | NAT2*5B |  |
| G | ITA | PAL33 | C | T | C | G | A | G | NAT2*4 | Intermediate |
| G | ITA |  | T | T | C | A | A | G | NAT2*6A |  |
| G | ITA | PAL34 | C | T | C | G | A | G | NAT2*4 | Intermediate |
| G | ITA |  | T | T | C | A | A | G | NAT2*6A |  |
| G | ITA | PAL35 | C | T | C | G | A | G | NAT2*4 | Intermediate |
| G | ITA |  | T | T | C | A | A | G | NAT2*6A |  |
| G | ITA | PAL36 | C | C | T | G | G | G | NAT2*5B | Slow |
| G | ITA |  | T | T | C | A | A | G | NAT2*6A |  |
| G | ITA | PAL37 | C | C | T | G | G | G | NAT2*5B | Slow |
| G | ITA |  | T | T | C | A | A | G | NAT2*6A |  |
| G | ITA | PAL38 | C | C | T | G | G | G | NAT2*5B | Slow |
| G | ITA |  | C | C | T | G | G | G | NAT2*5B |  |
| G | ITA | PAL39 | C | C | T | G | G | G | NAT2*5B | Slow |
| G | ITA |  | C | C | T | G | G | G | NAT2*5B |  |
| G | ITA | PAL40 | C | T | C | G | A | G | NAT2*4 | Intermediate |
| G | ITA |  | C | C | T | G | G | G | NAT2*5B |  |
| S | ITA | LAM1 | T | T | C | A | A | G | NAT2*6A | Slow |
| S | ITA |  | T | T | C | A | A | G | NAT2*6A |  |
| S | ITA | LAM10 | C | C | T | G | G | G | NAT2*5B | Slow |
| S | ITA |  | T | T | C | A | A | G | NAT2*6A |  |
| S | ITA | LAM4 | C | T | C | G | A | G | NAT2*4 | Fast |
| S | ITA |  | T | T | C | G | A | G | NAT2*13 |  |
| S | ITA | LAM5 | C | C | T | G | G | G | NAT2*5B | Slow |
| S | ITA |  | T | T | C | A | A | G | NAT2*6A |  |
| S | ITA | LAM6 | C | C | T | G | G | G | NAT2*5B | Slow |
| S | ITA |  | T | T | C | A | A | G | NAT2*6A |  |
| S | ITA | LAM7 | C | T | C | G | A | G | NAT2*4 | Intermediate |
| S | ITA |  | C | C | T | G | G | G | NAT2*5B |  |
| S | ITA | LAM8 | C | T | T | G | G | G | NAT2*12C | Intermediate |
| S | ITA |  | T | T | C | A | A | G | NAT2*6A |  |
| S | ITA | LAM9 | C | T | T | G | A | G | NAT2*11A | Intermediate |
| S | ITA |  | T | T | C | A | A | G | NAT2*6A |  |
| S | MOE | MOE1 | C | C | T | G | G | G | NAT2*5B | Slow |
| S | MOE |  | C | C | T | G | G | G | NAT2*5B |  |
| S | MOE | MOE10 | C | T | C | G | A | G | NAT2*4 | Fast |
| S | MOE |  | C | T | C | G | A | G | NAT2*4 |  |
| S | MOE | MOE11 | C | C | T | G | G | G | NAT2*5B | Intermediate |
| S | MOE |  | C | T | C | G | A | G | NAT2*4 |  |
| S | MOE | MOE13 | C | T | C | G | A | G | NAT2*4 | Intermediate |
| S | MOE |  | T | T | C | A | A | G | NAT2*6A |  |
| S | MOE | MOE14 | C | C | T | G | G | G | NAT2*5B | Slow |
| S | MOE |  | C | C | T | G | G | G | NAT2*5B |  |
| S | MOE | MOE15 | C | C | T | G | G | G | NAT2*5B | Slow |
| S | MOE |  | C | C | T | G | G | G | NAT2*5B |  |
| S | MOE | MOE16 | C | C | T | G | G | G | NAT2*5B | Slow |
| S | MOE |  | T | T | C | A | A | G | NAT2*6A |  |
| S | MOE | MOE17 | C | C | T | G | G | G | NAT2*5B | Slow |
| S | MOE |  | C | C | T | G | G | G | NAT2*5B |  |
| S | MOE | MOE18 | C | C | T | G | G | G | NAT2*5B | Slow |
| S | MOE |  | C | C | T | G | G | G | NAT2*5B |  |
| S | MOE | MOE3 | C | C | T | G | G | G | NAT2*5B | Intermediate |
| S | MOE |  | C | T | C | G | A | G | NAT2*4 |  |
| S | MOE | MOE6 | C | C | T | G | G | G | NAT2*5B | Intermediate |
| S | MOE |  | C | T | T | G | G | G | NAT2*12C |  |
| S | MOE | MOE7 | C | T | C | G | A | G | NAT2*4 | Intermediate |
| S | MOE |  | T | T | C | G | A | A | NAT2*7B |  |
| S | MOE | MOE8 | C | T | T | G | G | G | NAT2*12C | Fast |
| S | MOE |  | T | T | C | G | A | G | NAT2*13 |  |
| S | MOE | MOE9 | T | T | C | G | A | A | NAT2*7B | Slow |
| S | MOE |  | T | T | C | A | A | G | NAT2*6A |  |
| S | PER | PER46 | C | C | T | G | G | G | NAT2*5B | Slow |
| S | PER |  | C | C | T | G | G | G | NAT2*5B |  |
| S | PER | PER11 | C | T | C | G | A | G | NAT2*4 | Fast |
| S | PER |  | C | T | C | G | A | G | NAT2*4 |  |
| S | PER | PER12 | T | T | C | A | A | G | NAT2*6A | Slow |
| S | PER |  | T | T | C | A | A | G | NAT2*6A |  |
| S | PER | PER13 | C | C | T | G | G | G | NAT2*5B | Intermediate |
| S | PER |  | T | T | C | G | G | G | NAT2*12B |  |
| S | PER | PER15 | C | C | T | G | A | G | NAT2*5A | Intermediate |
| S | PER |  | C | T | C | G | A | G | NAT2*4 |  |
| S | PER | PER18 | C | C | T | G | G | G | NAT2*5B | Slow |
| S | PER |  | C | C | T | G | G | G | NAT2*5B |  |
| S | PER | PER22 | C | T | C | G | A | G | NAT2*4 | Fast |
| S | PER |  | T | T | C | G | A | G | NAT2*13 |  |
| S | PER | PER39 | C | C | T | G | G | G | NAT2*5B | Slow |
| S | PER |  | T | T | C | G | A | A | NAT2*7B |  |
| S | PER | PER40 | C | T | C | G | A | G | NAT2*4 | Fast |
| S | PER |  | C | T | C | G | A | G | NAT2*4 |  |
| S | PER | PER41 | C | T | C | G | G | G | NAT2*12A | Fast |
| S | PER |  | C | T | C | G | A | G | NAT2*4 |  |
| S | PER | PER42 | C | C | T | G | G | G | NAT2*5B | Intermediate |
| S | PER |  | C | T | C | G | G | G | NAT2*12A |  |
| S | PER | PER45 | C | C | T | G | G | G | NAT2*5B | Intermediate |
| S | PER |  | C | T | C | G | A | G | NAT2*4 |  |
| G | WAF | DEN4 | C | C | C | G | G | G | NAT2*5C | Slow |
| G | WAF |  | T | T | C | A | G | G | NAT2*6C |  |
| G | WAF | DEN5 | C | C | T | G | G | G | NAT2*5B | Slow |
| G | WAF |  | C | C | T | G | G | G | NAT2*5B |  |
| G | WAF | DEN22 | T | T | C | A | A | G | NAT2*6A | Slow |
| G | WAF |  | T | T | C | A | A | G | NAT2*6A |  |
| G | WAF | DEN27 | C | C | T | G | G | G | NAT2*5B | Slow |
| G | WAF |  | C | C | T | G | G | G | NAT2*5B |  |
| G | WAF | DEN28 | T | T | C | A | A | G | NAT2*6A | Slow |
| G | WAF |  | T | T | C | A | A | G | NAT2*6A |  |
| G | WAF | DEN30 | C | T | C | G | G | G | NAT2*12A | Fast |
| G | WAF |  | T | T | C | G | G | G | NAT2*12B |  |
| G | WAF | DEN31 | C | C | T | G | G | G | NAT2*5B | Slow |
| G | WAF |  | T | T | C | A | A | G | NAT2*6A |  |
| G | WAF | DEN32 | T | C | T | G | G | G | NAT2*5G | Slow |
| G | WAF |  | C | C | C | G | A | G | NAT2*5D |  |
| G | WAF | DEN33 | T | T | C | G | A | G | NAT2*13 | Fast |
| G | WAF |  | T | T | C | G | A | G | NAT2*13 |  |
| G | WAF | DEN48 | C | T | C | A | A | G | NAT2*6B | Slow |
| G | WAF |  | T | T | C | A | A | G | NAT2*6A |  |
| G | WAF | DEN49 | C | T | C | A | A | G | NAT2*6B | Intermediate |
| G | WAF |  | T | T | C | G | A | G | NAT2*13 |  |
| G | YKL | YM20 | C | T | C | G | A | G | NAT2*4 | Intermediate |
| G | YKL |  | T | T | C | A | A | G | NAT2*6A |  |
| G | YKL | YM21 | C | T | C | G | A | G | NAT2*4 | Fast |
| G | YKL |  | C | T | C | G | A | G | NAT2*4 |  |
| G | YKL | YM22 | C | C | T | G | G | G | NAT2*5B | Slow |
| G | YKL |  | T | T | C | G | A | A | NAT2*7B |  |
| G | YKL | YM23 | T | T | C | A | A | G | NAT2*6A | Slow |
| G | YKL |  | T | T | C | G | A | A | NAT2*7B |  |
| G | YKL | YM25 | C | T | C | G | A | G | NAT2*4 | Fast |
| G | YKL |  | C | T | C | G | A | G | NAT2*4 |  |
| S | YKL | YM10 | T | T | C | A | A | G | NAT2*6A | Slow |
| S | YKL |  | T | T | C | G | A | A | NAT2*7B |  |
| S | YKL | YM11 | C | C | T | G | G | G | NAT2*5B | Slow |
| S | YKL |  | T | T | C | A | A | G | NAT2*6A |  |
| S | YKL | YM12 | C | T | C | G | A | G | NAT2*4 | Intermediate |
| S | YKL |  | T | T | C | A | A | G | NAT2*6A |  |
| S | YKL | YM13 | C | T | C | G | A | G | NAT2*4 | Intermediate |
| S | YKL |  | T | T | C | G | A | A | NAT2*7B |  |
| S | YKL | YM14 | C | T | C | G | A | G | NAT2*4 | Fast |
| S | YKL |  | C | T | C | G | A | G | NAT2*4 |  |
| S | YKL | YM15 | C | C | T | G | A | G | NAT2*5A | Slow |
| S | YKL |  | T | T | C | A | A | G | NAT2*6A |  |
| S | YKL | YM16 | C | T | C | G | A | G | NAT2*4 | Intermediate |
| S | YKL |  | C | C | T | G | G | G | NAT2*5B |  |
| S | YKL | YM17 | C | T | C | G | A | G | NAT2*4 | Intermediate |
| S | YKL |  | C | C | T | G | G | G | NAT2*5B |  |
| S | YKL | YM18 | C | C | T | G | G | G | NAT2*5B | Slow |
| S | YKL |  | C | C | T | G | G | G | NAT2*5B |  |
| S | YKL | YM19 | C | T | C | G | A | G | NAT2*4 | Intermediate |
| S | YKL |  | T | T | C | A | A | G | NAT2*6A |  |
| S | YKL | YM2 | C | T | T | G | G | G | NAT2*12C | Intermediate |
| S | YKL |  | T | T | C | A | A | G | NAT2*6A |  |
