# Supplementary material for: Multiple Advantageous Amino Acid Variants in the NAT2 Gene in Human Populations
Source: PLoS One. 2008 Sep 5;3(9):e3136. doi: 10.1371/journal.pone.0003136 (PMC2527519; doi:10.1371/journal.pone.0003136)
Supplement: Table S3 — (0.04 MB DOC) [file pone.0003136.s006.doc]

Supplementary Table S3. Summary statistics of diversity of *NAT2* in the 9 population samples of the resequencing panel

| Population | N^1^ | Hd^2^ | Theta(pi)% | Theta(pi)%  (Syn) | Theta(pi)%  (NSyn) | Theta(s)% | Theta(s)%  (Syn) | Theta(s)%  (NSyn) | TD^3^ | Fay&Wu H |
| --- | --- | --- | --- | --- | --- | --- | --- | --- | --- | --- |
|  |  |  |  |  |  |  |  |  |  |  |
| Amhara | 12 | 0.409 | 0.234 | 0.412 | 0.183 | 0.190 | 0.334 | 0.148 | 0.868 | -1.909 |
| Egyptians | 20 | 0.647 | 0.263 | 0.461 | 0.205 | 0.161 | 0.284 | 0.126 | 1.922 | +0.168 |
| Italians | 16 | 0.767 | 0.277 | 0.522 | 0.206 | 0.173 | 0.304 | 0.135 | 1.990 | +0.833 |
| Greeks | 20 | 0.784 | 0.299 | 0.504 | 0.240 | 0.194 | 0.284 | 0.168 | 1.732 | +1.011 |
| Mordvin | 28 | 0.706 | 0.254 | 0.432 | 0.203 | 0.177 | 0.259 | 0.153 | 1.276 | +0.217 |
| Russians | 24 | 0.790 | 0.239 | 0.420 | 0.186 | 0.184 | 0.270 | 0.160 | 0.897 | +0.949 |
| Khanty&Mansi | 28 | 0.865 | 0.280 | 0.484 | 0.221 | 0.177 | 0.259 | 0.153 | 1.705 | +1.116 |
| Yakuts | 22 | 0.797 | 0.270 | 0.474 | 0.211 | 0.189 | 0.277 | 0.163 | 1.343 | +1.385 |
| Chukchee | 26 | 0.803 | 0.261 | 0.411 | 0.218 | 0.210 | 0.264 | 0.195 | 0.744 | +1.440 |

1. Number of gene copies; 2. Haplotype diversity; 3. Tajima's D
