# Supplementary material for: Multiple Advantageous Amino Acid Variants in the NAT2 Gene in Human Populations
Source: PLoS One. 2008 Sep 5;3(9):e3136. doi: 10.1371/journal.pone.0003136 (PMC2527519; doi:10.1371/journal.pone.0003136)
Supplement: Table S4 — (0.04 MB DOC) [file pone.0003136.s007.doc]

Supplementary Table S4. Pairwise linkage disequilibria between variable sites in the extended panel. D' (absolute values) below the diagonal; r^2^ above the diagonal. All P<0.0001

| Position | 282 | 341 | 481 | 590 | 803 | 857 |
| --- | --- | --- | --- | --- | --- | --- |
| 282 | - | .336 | .329 | .635 | .340 | .082 |
| 341 | .944 | - | .774 | .239 | .769 | .040 |
| 481 | .971 | .913 | - | .238 | .735 | .037 |
| 590 | .965 | .963 | 1.0 | - | .256 | .020 |
| 803 | .919 | .906 | .920 | .965 | - | .042 |
| 857 | .883 | 1.0 | 1.0 | 1.0 | 1.0 | - |
