# Supplementary material for: Multiple Advantageous Amino Acid Variants in the NAT2 Gene in Human Populations
Source: PLoS One. 2008 Sep 5;3(9):e3136. doi: 10.1371/journal.pone.0003136 (PMC2527519; doi:10.1371/journal.pone.0003136)
Supplement: Table S5 — (0.03 MB DOC) [file pone.0003136.s008.doc]

Supplementary Table S5. Allele frequencies at individual SNPs and associated Fst among 12 populations

| Position | Allele frequencies | | Fst |
| --- | --- | --- | --- |
| 282 | (C) .655 | (T) .345 | .000 |
| 341 | (C) .417 | (T) .583 | .026 |
| 481 | (C) .601 | (T) .399 | .021 |
| 590 | (A) .264 | (G) .736 | .014 |
| 803 | (A) .567 | (G) .433 | .016 |
| 857 | (A) .052 | (G) .948 | .041 |
