# Supplementary material for: Multiple Advantageous Amino Acid Variants in the NAT2 Gene in Human Populations
Source: PLoS One. 2008 Sep 5;3(9):e3136. doi: 10.1371/journal.pone.0003136 (PMC2527519; doi:10.1371/journal.pone.0003136)
Supplement: Table S6 — (0.08 MB DOC) [file pone.0003136.s009.doc]

Supplementary Table S6. Assignment of populations to nutritional categories. Population codes as in Supplementary Fig. S3.

| Code | Population | Nutritional  category | Ref. for DNA data |
| --- | --- | --- | --- |
|  |  |  |  |
| KUS | !Kung San | Hunter-gath. | Patin et al. 2006b |
| AMH | Amhara | Agriculturalist | This paper |
| ASK | Ashkenazi | Agriculturalist | Patin et al. 2006a |
| BKC | Baka Camerun | Hunter-gath. | Patin et al. 2006b |
| BKG | Baka Gabon | Hunter-gath. | Patin et al. 2006b |
| AKO | Bakola | . | Patin et al. 2006a |
| BED | Bedzan Pygmy | Hunter-gath. | Patin et al. 2006b |
| BIA | Biaka Pygmy | Hunter-gath. | Patin et al. 2006b |
| CHN | Chinese | Agriculturalist | Patin et al. 2006a |
| CHI | Chipewyan (Canada) | Hunter-gath. | Fuselli et al. 2007 |
| CHK | Chukchee | Hunter-gath. | This paper |
| CRE | Cree (Canada) | Hunter-gath. | Fuselli et al. 2007 |
| CZK | Czechs | Agriculturalist | This paper |
| WAF | Dendi | Agriculturalist | This paper |
| EGY | Egyptians | Agriculturalist | This paper |
| FRE | French | Agriculturalist | Patin et al. 2006a |
| GRE | Greeks | Agriculturalist | This paper |
| GUJ | Gujarati | Agriculturalist | Patin et al. 2006a |
| ITA | Italians | Agriculturalist | This paper |
| KZM | Kazakh | Pastoralist | Magalon et al. 2008 |
| K&M | Khanty&Mansi | Hunter-gath. | This paper |
| KYR | Kyrgyz | Pastoralist | Rabstein et al. 2006 |
| KRA | Kyrgyz (lowland) | Pastoralist | Magalon et al. 2008 |
| KRM | Kyrgys (highland) | Pastoralist | Magalon et al. 2008 |
| MAY | Maya (Mexico) | Agriculturalist | Fuselli et al. 2007 |
| MBU | Mbuti | Hunter-gath. | Patin et al. 2006b |
| MOE | Mordvins | Agriculturalist | This paper |
| MOR | Moroccans | Pastoralist | Patin et al. 2006a |
| NGC | Ngumba Cameroon | Agriculturalist | Patin et al. 2006b |
| ORO | Oromo | Pastoralist | This paper |
| P&C | Piapoco (Colombia) | Hunter-gath. | Fuselli et al. 2007 |
| ROM | Romanians | Agriculturalist | Rabstein et al. 2006 |
| PER | Russians | Agriculturalist | This paper |
| SAA | Saami | Hunter-gath. | Patin et al. 2006a |
| SAR | Sardinians | Agriculturalist | Patin et al. 2006a |
| SOM | Somali | Agriculturalist | Patin et al. 2006a |
| SUR | Surui (Brazil) | Hunter-gath. | Fuselli et al. 2007 |
| SWE | Swedes | Agriculturalist | Patin et al. 2006a |
| TJA | Tajik (Agalic) | Agriculturalist | Magalon et al. 2008 |
| TJU | Tajik (Urgut) | Agriculturalist | Magalon et al. 2008 |
| THA | Thai | Agriculturalist | Patin et al. 2006a |
| TUR | Turkmen | Pastoralist | Patin et al. 2006a |
| UZM | Uzbek | Pastoralist | Magalon et al. 2008 |
| XAV | Xavante | Hunter-gath. | Fuselli et al. 2007 |
| YKF | Yakut | Pastoralist | Fuselli et al. 2007 |
| YKL | Yakuts | Pastoralist | This paper |
| YOR | Yoruba (Nigeria) | Agriculturalist | Patin et al. 2006b |

Fuselli S, Gilman RH, Chanock SJ, Bonatto SL, De Stefano G, Evans CA, Labuda D, Luiselli D, Salzano FM, Soto G et al. 2007. Analysis of nucleotide diversity of NAT2 coding region reveals homogeneity across Native American populations and high intra-population diversity. Pharmacogenomics J 7:144-152

Magalon H, Patin E, Austerlitz F, Hegay T, Aldashev A et al., 2008 Population genetic diversity of the NAT2 gene supports a role of acetylation in human adaptation to farming in Central Asia. Eur J Hum Genet 16: 243-251.

Patin E, Barreiro LB, Sabeti PC, Austerlitz F, Luca F, Sajantila A, Behar DM, Semino O, Sakuntabhai A, Guiso N et al. 2006a. Deciphering the ancient and complex evolutionary history of human arylamine N-acetyltransferase genes. Am J Hum Genet 78:423-36.

Patin E, Harmant C, Kidd KK, Kidd J, Froment A, Mehdi SQ, Sica L, Heyer E, Quintana-Murci L. 2006b. Sub-Saharan African coding sequence variation and haplotype diversity at the NAT2 gene. Hum Mutat 27:720.

Rabstein S, Unfried K, Ranft U, Illig T, Kolz M, Rihs HP, Mambetova C, Vlad M, Bruning T, Pesch B. 2006. Variation of the N-acetyltransferase 2 gene in a Romanian and a Kyrgyz population. Cancer Epidemiol Biomarkers Prev 15:138-41.
